# Evaluation of Polysaccharide–Peptide Conjugates Containing the RGD Motif for Potential Use in Muscle Tissue Regeneration

**DOI:** 10.3390/ma15186432

**Published:** 2022-09-16

**Authors:** Marcin Kolasa, Katarzyna Czerczak, Justyna Fraczyk, Lukasz Szymanski, Slawomir Lewicki, Anna Bednarowicz, Nina Tarzynska, Dominik Sikorski, Grzegorz Szparaga, Zbigniew Draczynski, Szczepan Cierniak, Urszula Brzoskowska, Grzegorz Galita, Ireneusz Majsterek, Dorota Bociaga, Paulina Krol, Beata Kolesinska

**Affiliations:** 1 Military Institute of Hygiene and Epidemiology, Department of Pharmacology and Toxicology, Kozielska 4, 01-163 Warsaw, Poland; 2 Institute of Organic Chemistry, Faculty of Chemistry, Lodz University of Technology, Zeromskiego 116, 90-924 Lodz, Poland; 3 Department of Molecular Biology, Institute of Genetics and Animal Biotechnology, Polish Academy of Science, Postępu 36A, 05-552 Magdalenka, Poland; 4 Institute of Material Sciences of Textiles and Polymer Composites, Faculty of Material Technologies and Textile Design, Lodz University of Technology, Zeromskiego 116, 90-924 Lodz, Poland; 5 Military Institute of Medicine, Szaserow 128, 04-141 Warsaw, Poland; 6 Department of Clinical Chemistry and Biochemistry, Medical University of Lodz, Narutowicza 60, 90-136 Lodz, Poland; 7 Institute of Materials Science and Engineering, Lodz University of Technology, Stefanowskiego 1/15, 90-537 Lodz, Poland; 8 Lukasiewicz Research Network-Textile Research Institute, Brzezinska 5/15, 92-103 Lodz, Poland

**Keywords:** polysaccharide–peptide conjugates, alginate, chitosan, ternary mixtures of polysaccharide nonwoven material, biological activity, antibacterial activity, regenerative medicine

## Abstract

New scaffold materials composed of biodegradable components are of great interest in regenerative medicine. These materials should be: stable, nontoxic, and biodegrade slowly and steadily, allowing the stable release of biodegradable and biologically active substances. We analyzed peptide-polysaccharide conjugates derived from peptides containing RGD motif (H-RGDS-OH (**1**), H-GRGDS-NH_2_ (**2**), and cyclo(RGDfC) (**3**)) and polysaccharides as scaffolds to select the most appropriate biomaterials for application in regenerative medicine. Based on the results of MTT and Ki-67 assays, we can state that the conjugates containing calcium alginate and the ternary nonwoven material were the most supportive of muscle tissue regeneration. Scanning electron microscopy imaging and light microscopy studies with hematoxylin–eosin staining showed that C2C12 cells were able to interact with the tested peptide–polysaccharide conjugates. The release factor (Q) varied depending on both the peptide and the structure of the polysaccharide matrix. LDH, Alamarblue^®^, Ki-67, and cell cycle assays indicated that peptides **1** and **2** were characterized by the best biological properties. Conjugates containing chitosan and the ternary polysaccharide nonwoven with peptide **1** exhibited very high antibacterial activity against *Staphylococcus aureus* and *Klebsiella pneumoniae*. Overall, the results of the study suggested that polysaccharide conjugates with peptides **1** and **2** can be potentially used in regenerative medicine.

## 1. Introduction

Skeletal muscles are one of the most abundant tissues in the human body. They constitute about 40–45% of the total body weight and are essential for various functions [1,2]. These muscles, to some extent, can regenerate lost tissue after injury [3]. However, if the volumetric muscle loss (VML) is very high, the remaining muscle tissue cannot completely regenerate without medical intervention [4,5,6]. This can have a significant effect on the quality of life and reduce patients’ mobility [7]. It is estimated that only up to 20% of muscle mass can be restored due to the adaptability and regenerative capacity of muscles. Skeletal muscle injuries that cannot be repaired by natural regenerative properties are often caused by road accidents, explosions, combat, and sports, all of which lead to acute loss of muscle tissue [8]. Around 35–55% of sports injuries involve muscle damage [9] and require reconstructive surgery if over 20% of the muscle mass is damaged [10]. Additionally, some patients may experience progressive muscle loss if they are affected by metabolic disorders or inherited genetic diseases such as Duchenne muscular dystrophy, amyotrophic lateral sclerosis, or Charcot–Marie–Tooth disease (in children) [11,12,13,14]. Other causes of muscle atrophy are peripheral nerve injury, chronic kidney disease, diabetes, malnutrition, cancer cachexia, bacterial infections, and heart failure [15,16].

Muscle regeneration involves a heterogeneous population of satellite cells, interstitial cells, and blood vessels and is mainly regulated by proteins and secreted factors of the extracellular matrix (ECM) [17,18]. However, in cases of VML, the regenerative capacity of skeletal muscles is reduced due to the physical removal of the essential factors of regeneration from the body [19]. The balance between protein synthesis and degradation is also disturbed [20], which leads to the activation of degradation pathways such as proteasome and autophagosome pathways. This results in muscle atrophy, accompanied by a gradual decrease in muscle mass and the diameter of muscle fibers [21,22]. The clinical therapies that are currently available for the treatment of muscle atrophy include free functional muscle transfer; however, this has side effects and does not guarantee complete restoration of muscle functions to the preinjury state [23].

Tissue engineering and regenerative medicine are promising therapeutic approaches for patient-specific musculoskeletal treatment that can restore muscle function, stimulate regeneration, and improve the quality of life [24]. Skeletal muscle engineering is of two basic types [25]. In vitro tissue engineering develops tissue structures displaying similar structural and functional properties as those of native tissue prior to the transplant. However, a prerequisite for this approach is the differentiation of skeletal muscle myoblasts or muscle precursors into multinucleated myotubes [26]. On the other hand, the second approach, in situ tissue engineering, focuses on constructing materials for the delivery of cells and/or inducing factors that are used for subsequent remodeling in the host environment.

Biomaterials with tailored properties are often applied in both in vitro tissue engineering and in vivo regeneration. The materials used for musculoskeletal engineering should improve cell and tissue function by modulating cell adherence, survival, organization, and differentiation. Some of the commonly used biomaterials are ECM derivatives, such as collagen [27,28,29], fibrin [30], gelatin [31], and keratin [32], as well as polysaccharides such as hyaluronic acid [33], chitosan [34], and alginate [35]. Natural biomaterials are more advantageous than synthesized ones as they are biocompatible and enzymatically degradable and also contain functional groups that facilitate the attachment of small molecules and growth factors. However, these materials exhibit inter-batch variability and, in some cases, immunogenicity, which are major disadvantages [36]. On the other hand, the mechanical properties and chemical compositions of synthetic polymers can be controlled such that they more closely resemble those of naturally derived biomaterials [37]. Synthetic polymers widely used in musculoskeletal tissue engineering include poly-L-lactic acid, poly (lactic-co-glycolic acid), and polycaprolactone [37,38,39,40,41]. However, these materials also have disadvantages, such as limited cell adhesion, and their degradation products tend to impede muscle regeneration and promote inflammatory responses [42].

In order to facilitate the use of natural and synthetic polymers as scaffolds for musculoskeletal tissue, their properties should be improved or modified by introducing bioactive molecules. Growth factors are one of the most commonly used bioactive molecules. Insulin-like growth factor [43,44], vascular endothelial growth factor [45,46,47,48], fibroblast growth factors [49], hepatocyte growth factor [50,51], and stromal cell-derived factor-1 [52,53,54] are a few examples of growth factors used in the regeneration of muscle tissue.

A key characteristic of biomaterials used in regenerative medicine is their ability to influence the growth, adhesion, and proliferation of endogenous cells. One way to ensure cell adhesion to the material is to modify it so that it contains a peptide or a protein fragment with a sequence matching a specific receptor. Two of the most common and simplest peptide sequences that bind to cells are RGD and RGDS [55]. RGD is a structural motif present in ECM proteins, such as fibronectin, vitronectin, fibrinogen, osteopontin, and bone sialoprotein, which ensures binding to integrins [56,57]. This motif is also found in some laminins and collagens [58]. Compared to native ECM proteins, RGD peptides minimize the risk of an immune response or pathogen transmission, especially when protein xenografts are isolated from other organisms. Because RGD peptides act as ligands for integrin receptors, they have an impact on cellular adhesion and proliferation. Furthermore, these peptides can be easily synthesized and are inexpensive, and thus can be applied in clinical practice. Another important advantage of RGD peptides is that they can be coupled to surfaces with controlled densities and orientations and hence more frequently applied as additives to modify and improve the properties of materials used in regenerative medicine [59,60,61,62,63].

In this study, we tested the applicability of peptide–polysaccharide non-covalent conjugates (obtained by dip-coating approach) as materials for muscle tissue regeneration. The following RGD motif-containing peptides were analyzed: H-RGDS-OH (**1**), H-GRGDS-NH_2_ (**2**), and cyclo(RGDfC) (**3**). In addition, three polysaccharide nonwovens based on calcium alginate (matrix **A**), chitosan (matrix **B**), and a mixed, ternary nonwoven material containing calcium alginate, chitosan, and chitin butyryl-acetyl co-polyester (BAC 90:10) in equal amounts (matrix **C**) were tested as scaffolds. All these polysaccharides have previously been proven to promote tissue regeneration and undergo biodegradation. We expected that the material containing equal amounts of negatively charged calcium alginate, positively charged chitosan, and neutral BAC 90:10 would have different properties compared to those of its individual constituents. Furthermore, we assumed that the presence of the relatively hydrophobic BAC 90:10 would have a positive impact on the biological activity of the final product and therefore improve its utility in regenerative medicine. It was also hypothesized that chitosan-based materials are characterized by antimicrobial activity. We investigated the biological effects of peptides containing the RGD motif and with diverse structures (peptides with linear and cyclic structures, as well as with an amide and carboxylic bond at the C-terminus). Additionally, we examined the influence of the structure of the polysaccharide matrix on the biological activity of the tested conjugates. We attempted to verify if the final peptide–polysaccharide conjugates have appropriate biological properties, well as limited cytotoxicity and ability to promote cellular proliferation and differentiation, which would make them suitable for use in regenerative medicine. Additionally, preliminary attempts were made to evaluate the basic mechanical and adhesive properties. It was crucial to see if the RGD peptides released from the polysaccharide matrices would affect the utility of the conjugates as materials useful in regenerative medicine.

## 2. Materials and Methods

### 2.1. Synthesis of Peptides Containing the RGD Motif

Peptides H-RGDS-OH (**1**), H-GRGDS-NH_2_ (**2**), and cyclo(RGDfC) (**3**) were synthesized on chlorotrityl resin (peptide **1** and linear precursor of **3**) or Rink Amide resin (peptide **2**) using the Fmoc/tBu strategy. Peptides **1** and **2**, as well as the linear precursor of **3**, were prepared according to the standard procedure used for the synthesis of solid-phase peptides. Cyclic peptide **3** was obtained by applying the standard solution used for the synthesis of cyclic peptides, using a high-dilution approach [64]. 4-(4,6-Dimethoxy-1,3,5-triazin-2-yl)-4-methylmorpholinium toluene-4-sulfonate (DMT/NMM/TosO^−^) [65] was used as a coupling reagent in the synthesis of both linear peptides and cyclic peptide (details of the synthetic protocol and high-performance liquid chromatography (HPLC) and mass spectrometry (MS) spectra are presented in Figures S1–S8 in the Supplementary Materials).

For the determination of peptide purity, an LC Dionex UltiMate 3000 system (Thermo Fisher Scientific, Waltham, MA, USA) with a Kinetex C18 column (100 × 4.6 mm) and a gradient program based on H_2_O (B) or CH_3_CN (A) with 0.1% trifluoroacetic acid (TFA) was used. The peptides were analyzed at wavelengths 220 and 254 nm. The parameters applied for preparative HPLC were as follows: CombiFlash, EZPrep, Teledyne ISCO (Lincoln, NE, USA); Supelco Discovery BIO Wide Pore C18 column (25 cm × 21.2 mm, 10 mm; Sigma-Aldrich, St. Louis, MO, USA); flow rate 5 mL/min; detection wavelengths 220 and 254 nm; gradient A and B. MS analysis was performed on a Bruker microOTOF-QIII system (Bruker Corporation, Billerica, MA, USA).

#### 2.1.1. Synthesis of H-RGDS-OH (**1**)

The starting materials used for each coupling were chlorotrityl resin (1.0 g, 1.0 mmol), Fmoc-Ser(tBu)-OH (1.151 g, 3.0 mmol), Fmoc-Asp(OtBu)-OH (1.234 g, 3.0 mmol), Fmoc-Gly-OH (0.892 g, 3.0 mmol), Fmoc-Arg(Pbf)-OH (1.946 g, 3.0 mmol), DMT/NMM/TosO^−^ (1.239 g, 3.0 mmol), and N-methylmorpholine (NMM) (0.66 mL, 6.0 mmol). The synthesized peptide was cleaved from the resin using a mixture of TFA, H_2_O, and triisopropylsilane (TIS) (95:2.5:2.5, *v*:*v*:*v*). HPLC (15–95% A in 30 min): t*_R_* 2.85 min, purity = 95%. MS: 434.2295 ([M + H]^+^, C_15_H_28_N_7_O_8_^+^; calc. 433.19).

#### 2.1.2. Synthesis of H-GRGDS-NH_2_ (**2**)

The starting materials used for each coupling were Rink Amide resin (1.0 g, 0.7 mmol), Fmoc-Ser(tBu)-OH (0.805 g, 2.1 mmol), Fmoc-Asp(OtBu)-OH (0.864 g, 2.1 mmol), Fmoc-Gly-OH (0.624 g, 2.1 mmol), Fmoc-Arg(Pbf)-OH (1.362 g, 2.1 mmol), Fmoc-Gly-OH (0.624 g, 2.1 mmol), DMT/NMM/TosO^−^ (0.903 g, 2.1 mmol), and NMM (0.46 mL, 4.2 mmol). The synthesized peptide was cleaved from the resin using a mixture of TFA, H_2_O, and TIS (95:2.5:2.5, *v*:*v*:*v*). HPLC (15–95% A in 30 min): t*_R_* 2.34 min, purity = 98%. MS: 490.2285 ([M + H]^+^, C_17_H_31_N_9_O_8_^+^; calc. 489.23).

#### 2.1.3. Synthesis of cyclo(RGDfC) (**3**)

The linear precursor H_2_N-Asp(OtBu)D-Phe-Cys(Trt)-Arg(Pbf)Gly-OH was used. The starting materials used for each coupling were chlorotrityl resin (1.0 g, 1.0 mmol), Fmoc-Gly-OH (0.892 g, 3.0 mmol), Fmoc-Arg(Pbf)-OH (1.946 g, 3.0 mmol), Fmoc-Cys(Trt)-OH (1.757 g, 3.0 mmol), Fmoc-D-Phe-OH (1.162 g, 3.0 mmol), Fmoc-Asp(OtBu)-OH (1.234 g, 3.0 mmol), DMT/NMM/TosO^−^ (1.239 g, 3.0 mmol), and NMM (0.66 mL, 6.0 mmol). The synthesized peptide was cleaved from the resin using 50% TIS in dichloromethane. HPLC (15–95% A in 30 min): t*_R_* 25.7 min, purity = 98%. MS: 1147.4739 ([M + H]^+^, C_60_H_74_N_8_O_12_S_2_^+^; calc. 1146.45).

Cyclization was performed as follows. To a solution of N,N-diisopropylethylamine (DIPEA) (162 µL, 0.9 mmol) in dichloromethane (200 mL), 1-[bis(dimethylamino)methylene]-1H-1,2,3-triazolo[4,5-b]pyridinium 3-oxide hexafluorophosphate (HATU) (114 mg, 0.3 mmol) with hydroksybenzotriazol (HOBt) (40.5 mg, 0.3 mmol), the linear precursor (350 mg, 0.3 mmol), and DIPEA (54 µL) were added dropwise for 2 h. The solution was vigorously stirred and left for 24 h. After complete conversion of the peptide, the mixture was concentrated to 50 mL. The solution was washed with water (30 mL), 1 M NaHSO_4_ (30 mL), water (30 mL), 1 M NaHCO_3_ (30 mL), and once again with water (30 mL). After removal of the organic solvent, deprotection was performed analogously to the standard procedure to allow cleavage of the peptide from the resin. HPLC (15–95% A in 30 min): t*_R_* 3.65 min, purity = 97%. MS: 579.2256 ([M + H]^+^, C_24_H_34_N_8_O_7_S^+^; calc. 578.23).

### 2.2. Preparation of Nonwovens **A**, **B**, and **C** Based on Polysaccharides

Calcium alginate (**A**) with a surface mass of 98.8 g/m^2^ and chitosan (**B**) with a surface mass of 116 g/m^2^ were prepared as described previously [66]. In short, manufacture nonwovens was performed in the laboratory roller carding ma-chine with an elastic covering, Befama (BEFAMA Sp. z o.o., Kalina, Poland) equipped with an Asselin (ANDRITZ Asselin-Thibeau S.A.S., Elbeuf sur Seine, France) horizontal stacker. Needle punching of the fleece was carried out on an Asseline needle punching machine (ANDRITZ Asselin-Thibeau S.A.S., Elbeuf sur Seine, France) with an upper needle plate. The technological parameters of needle punching were as follows—type of needles—15 × 18 × 40 × 3 ½ RB (Groz-Beckert^®^, Albstadt-Ebingen, Germany); needle density 9.5; number of needle punches 60/cm^2^; depth of needle punching 12 mm.

The mass per square meter of nonwovens was determined according to the ISO 9073-1:1989 standard.

To obtain a ternary nonwoven **C** composed of calcium alginate–chitosan–chitin butyryl-acetyl co-polyester (BAC 9:1), the following fibers were mixed at a 1:1:1:ratio: (a) calcium alginate fibers prepared using the wet solution method developed at the Institute of Material Sciences of Textiles and Polymer Composites, Lodz University of Technology [67]; (b) chitosan fibers prepared using the wet-spinning solution method developed by Wawro and Pighinelli [68]; and (c) BAC 9:1 fibers prepared using the method developed at the Lodz University of Technology and implemented at Tricomed SA [69,70,71].

Calcium alginate fibers were prepared by adding sodium alginate Protanal LF 10/60LS (FMC Biopolymer, Sandvika, Norway) and coagulating solvent CaCl_2_. The final 2-dtex fibers were cut to 38 mm.

Chitosan fibers were prepared using chitosan (Primex Co., Myre, Norway) with a molecular weight of 350 kDa and 90% degree of deacetylation (based on Fourier-transform infrared spectroscopy (FTIR)). The final 2.2-dtex fibers were cut to 37.1 mm.

BAC 9:1 fibers were prepared using chitin (Mahtani Chitosan Pvt. Ltd., Veraval, India) with a viscosimetric molecular weight of 452.1 × 103 g/mol and 96.0% degree of deacetylation (based on FTIR). The fibers were spun from a 15% ethanol solution into a water coagulation bath containing 5% ethanol. The final 2.5-dtex fibers were cut to 38 mm.

#### Formation of Nonwoven **C** from Alginate/Chitosan/BAC 9:1 Fibers

Nonwoven **C** containing alginate/chitosan/BAC 9:1 fibers were obtained using the dry-laid method, which involves carding and subsequent web bonding by needle-punching. The nonwoven fleece was formed on a Befama (Kalna, Poland) carding machine. The fibers were mixed during carding. The carding machine was fed a third of each fiber type, with 400 g/m^2^ of the feeding conveyor. After carding, the fleece was transported to the stacker and then to the needler for needling at a depth of 12 mm (with 40 needles/cm^2^). The weight of the nonwoven was 120 g/m^2^. The characteristics of the applied fibers and the prepared polysaccharide nonwovens are presented in Table 1 and Table 2.

The unmodified and modified nonwovens were observed under a scanning electron microscope (NOVA NanoSEM 230; FEI, Hillsboro, OR, USA) at a magnification of 500× to assess their surface morphology and structures.

### 2.3. Synthesis of Conjugates **A1**–**A3**, **B1**–**B3**, and **C1**–**C3** of Polysaccharide Nonwovens **A**–**C** Modified with Peptides **1**–**3** Containing the RGD Motif

Polysaccharide nonwovens **A**–**C** with a dimension of 25 cm^2^ were used to obtain polysaccharide–RGD peptide conjugates. Briefly, a solution of 25 mg of RGD motif-containing peptides **1**–**3**, dissolved in 15 mL of ethanol and 5 mL of water, was applied to each nonwoven. Then, polysaccharide–peptide conjugates with a peptide concentration of 1 mg/cm^2^ were obtained. The nonwoven was immersed in the peptide solution (dip-coating) for 5 min. After the peptide solution was applied to the nonwovens, the final materials were dried at 35 °C. The remaining solutions were subjected to HPLC. Residual peptides constituted less than 1% of the total weight (based on the peak area). Considering the mass per unit area of the used polysaccharide nonwovens, the percentage content of the peptides on the polysaccharide matrices was estimated at 9% (*w*/*w*) for nonwoven **A**, 8% (*w*/*w*) for nonwoven **B**, and 8% (*w*/*w*) for nonwoven **C**. A total of nine polysaccharide nonwovens (**A1**–**A3**, **B1**–**B3**, **C1**–**C3**) modified with RGD motif-containing peptides were obtained (Table S1 in the Supplementary Materials).

### 2.4. Biological Research

#### 2.4.1. Cell Culture

The C2C12 cell line (ATCC^®^ CRL-1772™) from the ATCC collection (Manassas, VA, USA) was used for the analysis. Cells were cultured in Dulbecco’s Modified Eagle Medium (DMEM) supplemented with 10% fetal bovine serum and antibiotics (50 U/mL of penicillin and 50 µg/mL of streptomycin; Life Technologies, Warsaw, Poland) under aseptic-standard conditions (37 °C, 95% humidity, 5% CO_2_). Cells were grown in continuous cultures and passaged 4–12 times with 0.25% trypsin (Life Technologies, Warsaw, Poland) after reaching 80–90% confluence. Then, cells were thawed from the cell bank and passaged twice. Following trypsinization, cells were centrifuged at 400× *g* for 5 min. The resulting cell pellets were resuspended in 1 mL of fresh media and counted (ADAM MC Automated Mammalian Cell Counter; Digital Bio, Seoul, Korea).

#### 2.4.2. Cytotoxicity of Polysaccharide Conjugates **A**–**C** with Peptides **1**–**3**

MTT (3-(4,5-dimethylthiazol-2-yl)-2,5-diphenyltetrazolium bromide) test was performed using the C2C12 myoblast cell line (ATCC^®^ CRL-1772™; (ATCC, Manassas, VA, USA). Briefly, Polysaccharide conjugates **A**–**C** with peptides **1**–**3** at (2 cm^2^ of modified non-woven) were incubated in DMEM (2 mL) (Corning) for 48 h at 37 °C to attain extracts. Then, cells were seeded in a 96-well plate at a density of 1 × 10^4^ cells/well and grown at 37 °C (5% CO_2_). At 24 h, the culture medium was replaced by extract solutions. After 24 or 72 h, the culture medium was removed, and 1 mg/mL of MTT was added. Following a 2 h incubation, the MTT solution was discarded, and 100 µL of isopropanol (Sigma Aldrich, Poznan, Poland) was added. The absorbance of the culture was read at 570 and 650 nm using a microplate reader (Victor X4 Perkin Elmer; PerkinElmer, Inc., Waltham, MA, USA). Cells grown for 48 h in DMEM were used as a negative control, while those treated with dimethyl sulfoxide (DMSO) were used as a positive control. All experiments were performed in triplicate. The results are expressed as a percentage of the negative control with the standard deviation (SD). Statistical significance was assessed using a one-way analysis of variance (ANOVA) and assumed at *** *p* < 0.001, ** *p* < 0.01, and * *p* < 0.05.

#### 2.4.3. Cell Proliferation (Ki-67 Assay) of Polysaccharide Conjugates **A**–**C** with Peptides **1**–**3**

Polysaccharide conjugates **A**–**C** with peptides **1**–**3** at (2 cm^2^ of modified non-woven) were incubated in DMEM (2 mL) (Corning) for 48 h at 37 °C to attain extracts. Then, cells were seeded in a 24-cell plate (2 × 10^4^ cells/well). After 24 h, cells were washed twice with phosphate-buffered saline (PBS), and the culture medium was replaced by extract solutions. After 24 h or 72 h, the culture medium was removed, and cells were collected (0.25% trypsin, 5–7 min, 37 °C) and flushed twice with PBS. Then, cells were resuspended in 1 mL of cold 70% ethanol/PBS solution (4 °C) and stored in a freezer (−20 °C) for 1−30 days. On the day of the analysis, cells were centrifuged (300× *g*, 5 min) and stained with Ki67, as per the manufacturer’s protocol. For Ki-67 staining, 10 µL of mouse anti-Ki67 antibody (BD Bioscience, Warsaw, Poland) in 200 µL of PBS was applied for 30 min at room temperature, and then cells were flushed twice with a PBS solution. The proliferation assay was performed using a flow cytometer (FACS Calibur; BD, San Jose, CA, USA). For this purpose, the cell cycle analysis was stopped after 10,000 cells, and acquisition was stopped after 25,000 cells, as calculated using the FCS Express program (De Novo Software, Pasadena, CA, USA). The results are presented as mean ± SD from three independent experiments (*n* = 9).

#### 2.4.4. Sample Preparation for Microscopic Examination

For microscopic analysis, nonwovens **A1**–**A3**, **B1**–**B3**, and **C1**–**C3** were cut into 4 cm^2^ samples and folded to fit the cell culture inserts (Falcon). The inserts containing the samples were placed in a 12-well culture plate, and 100 μL of C2C12 cell suspension (1.0 × 10^6^ cells/mL) was seeded onto the scaffolds in duplicate. Then, 3 mL of culture medium was added to each well and 0.3 mL of culture medium to the insert. Cells were cultured for 24 h under standard conditions.

- Sample preparation for scanning electron microscopy (SEM) imaging

After cell culture, samples of nonwovens with cells were fixed in 4% paraformaldehyde (Thermo Fisher Scientific) for 24 h at 4 °C. Then, samples were washed twice with a Ca/Mg-free PBS solution (Sigma-Aldrich) and deionized water. Subsequently, samples were dehydrated using graded ethanol solutions (30–100%). For complete removal of water, polysaccharide nonwovens **A1, B1,** and **C1** with cells were dried in a critical point dryer (LEICA EM CPD300; Leica, Wetzlar, Germany). Finally, samples were sputter-coated with 10 nm of carbon (SafeMatic CCU-010 LV Compact Coating Unit; SafeMatic, Zizers, Switzerland) and imaged under a scanning electron microscope (JSM-6610LV; JEOL, Peabody, MA, USA).

- Sample preparation for optical microscopy imaging

Samples of nonwovens with cells were frozen at −20 °C and cut into 8 nm slices on a CRYO-2000 cryostat (Sakura Finetek, Alphen an den Rijn, The Netherlands). Specimens were stained with hematoxylin and eosin according to the standard procedure. Then, imaging was performed under a PANNORAMIC Diagnostic Scanners microscope (3DHISTECH, Budapest, Hungary) at a magnification of 400×.

#### 2.4.5. Antibacterial Properties of the Conjugates of Polysaccharides **A**–**C** with Peptides **1**–**3**

Conjugates of polysaccharide nonwovens **A**–**C** with peptides **1**–**3** were assessed for their antibacterial properties with unmodified polysaccharide nonwovens **A**–**C** as a control. Six samples of each tested conjugate and control material were used for the analysis. The conjugates were tested according to the ISO 20743:2013 standard. Control samples were placed in separate sterile containers and treated with 70% ethanol for 30 min prior to the test. The conjugates (**A1**, **A2**, **A3**, **B1**, **B2**, **B3**, **C1**, **C2**, **C3**), as well as the unmodified nonwovens (**A**–**C**), were further sterilized. Each side of the sample was irradiated with UV-C light for 30 min. *Staphylococcus aureus* ATCC 6538 and *Klebsiella pneumoniae* ATCC 4352 (Manassas, VA, USA) were used as bacterial test strains.

Suspensions with density varying between 1.0 × 10^5^ and 3.0 × 10^5^ CFU/mL were prepared for each of the strains. Test samples were inoculated with 0.2 mL of the bacterial suspension. After inoculation, 20 mL of neutralizer was added to the tested conjugates and the control materials. In order to determine the initial number of bacteria per sample, half of the samples (including both the controls and conjugates) were seeded on tryptone soya agar (TSA) medium at 100–10^−4^ dilutions. The remaining samples were incubated at 37 ± 2 °C for 24 h. After incubation, another 20 mL of neutralizer was added, and samples were seeded on the TSA medium at 100–10^−4^ dilutions to determine the final count of bacteria per sample.

#### 2.4.6. Release of Peptides **1**–**3** from **A1**–**A3**, **B1**–**B3**, and **C1**–**C3** Conjugates

In order to evaluate the release of peptides from nonwoven substrates, a pharmaceutical availability study was carried out using the modified basket method (the samples used are listed in Table S2 in the Supporting Material). The content of peptides **1**–**3** in the nonwovens **A**–**C** was 2 mg/cm^2^. Phosphate buffer (PBS 1× concentration) at 37 °C was used as an acceptor fluid. The sample was washed with this fluid at a constant speed of 240 rpm. A fixed modulus maintained the ratio of sample mass and acceptor fluid volume at 1:1000. Samples with dimensions of approximately 2 × 2 cm and a modulus of 1:1000 were flooded with the acceptor fluid at 37 °C and maintained at a constant temperature of 37 °C in a water bath. Then, samples were collected at the following time intervals: 30 s, 30 min, 1 h, 1 h 30 min, 2 h, 2 h 30 min, 3 h, 4 h, 5 h, 6 h, and 24 h. The amount of acceptor fluid was kept constant, and the amount of fluid was topped up by the amounts taken for testing. Solutions containing 7.99, 8.13, and 4.89 mg of peptides **1**, **2**, and **3** in 5 mL of PBS were used to obtain six diluted solutions. The diluted solutions were examined spectrophotometrically at a wavelength range of 190–660 nm using Thermo Scientific Orion AquaMate 8000 UV–VIS spectrophotometer (Thermo Fisher Scientific, Waltham, MA, USA) with VISION Lite Scan software. In order to evaluate the efficiency of the release of peptides, the absorbance values were determined at 202.5, 202.6, and 202.4 nm for peptides **1**, **2**, and **3**, respectively (details on how the standard curves were determined are presented in Table S3 and Figure S12 in the Supporting Material). Based on the absorbance measurements, the concentrations of active substances were calculated from the calibration equation, and then the percentages of active substances released (release factor Q) were determined. Additionally, in order to eliminate the influence of the degradation of polysaccharide matrices (formation of water-soluble sodium alginate or soluble chitosan salts), studies were carried out on the loss of conjugate mass during incubation. Weights of incubated samples (after drying) were determined for all **A1**–**A3**, **B1**–**B3**, and **C1**–**C3** conjugates at all measurement points (all times).

#### 2.4.7. Effects of Peptides **1**–**3** on C2C12 Cells

- Alamarblue^®^ and LDH assays

The Alamarblue^®^ assay was performed as previously described [72]. After trypsinization (0.25% trypsin, 5–7 min, 37 °C), cells were seeded in a 96-cell culture plate at a density of 1.5 × 10^3^ cells/well. After 24 h, cells were washed twice with PBS (Life Technologies, Warsaw, Poland), and a fresh medium was added to the wells. Next, peptides **1**–**3** at final concentrations of 0 (control), 0.01, 0.05, 0.1, 0.5, or 1 mM were added. Alamarblue^®^ (Life Technologies, Warsaw, Poland) was added directly to the wells at 10% of the volume. Fluorescence (excitation 544 nm, emission 590 nm) was measured after 24, 48, and 72 h of incubation using a Fluostar Omega microplate reader (FLUOstar Omega; BMG Labtech, Ortenberg, Germany). The results are presented as mean ± SD of three independent experiments (*n* = 12). The LDH assay was also conducted as previously described [73]. Briefly, after trypsinization (0.25% trypsin, 5–7 min, 37 °C), cells were seeded in a 96-cell culture plate at a density of 1.5 × 10^3^ cells/well. After 24 h, cells were washed twice with PBS (Life Technologies, Warsaw, Poland), and a fresh medium was added to the wells. Next, peptides **1**–**3** with final concentrations of 0 (control), 0.01, 0.05, 0.1, 0.5, or 1 mM were added. After 24 and 72 h, 150 µL of the supernatant was collected and stored at −8 °C. The activity of the lactate dehydrogenase (LDH) enzyme released from the cells was assessed using the Pierce LDH Cytotoxicity Assay Kit (Life Technologies, Warsaw, Poland). The absorbance of the sample was measured at 540 nm with the background cutoff at 490 nm (FLUOstar Omega; BMG Labtech GmbH, Ortenberg, Germany). The results are presented as the percentage of the control values (mean ± SD). Each assay was performed in triplicate (*n* = 12).

- Genotoxicity analysis

Cells were incubated in 12-well plates (2 × 10^5^/well) and cultured in 1 mL of complete Eagle’s Minimum Essential Medium for 24 h. Untreated cells containing resazurin solution (10% of the cell culture volume) were used as a negative control, while cells incubated with 10% DMSO were used as a positive control. After cell adhesion, cells were incubated with active peptides for 48 h. The contents of the wells were removed, 0.3 mL of accutase was added to each well, and cells were harvested. Then, harvested cells were centrifuged and suspended in 0.37% low-melting-point (LMP) agarose. The obtained pellet was suspended in 0.37% LMP agarose and applied to slides coated with LMP agarose. Samples were incubated with the lysis buffer containing 1% Triton X-100 (pH 10) for 1 h at 4 °C. After lysis, cells were incubated for 20 min at 4 °C and then electrophorized (17 V, 32 mA, 20 min). Then, slides were rinsed three times with distilled water and completely dried. Samples were stained with DAPI (4,6-diamidino-2-phenylindole) fluorescent dye and visualized under a fluorescence microscope. Statistical analysis was performed using the Sigma Plot program (Systat Software, Inc., Chicago, IL, USA). Additionally, normality was tested using the Shapiro–Wilk test. Because the analysis of cell viability indicated normal data distribution, statistical analysis was performed using the Student’s *t*-test. As the Comet assay revealed nonparametric distribution, statistical analysis of two groups was performed using the Mann–Whitney rank-sum test. Each assay was performed in triplicate. * *p* < 0.05, ** *p* < 0.01, and *** *p* < 0.001 were considered statistically significant.

- Cell viability, apoptosis, and necrosis analysis

Cell viability was assessed using a previously described procedure [74]. Briefly, The C2C12 (ATCC^®^ CRL-1772™) from the ATCC collection (Manassas, VA, USA) cells were trypsinized (0.05% trypsin, 5–7 min, 37 °C) and seeded in a 48-cell culture plate at a density of 5 × 10^3^ cells/well. After 24 h, cells were washed twice with a PBS solution. Then, fresh culture medium was added and peptides **1**–**3** at final concentrations of 0 (control), 0.01, 0.05, 0.1, 0.5, or 1 mM were applied. After 24 or 72 h of floating, cells were collected, and those adhered were trypsinized (0.25% trypsin, 5–7 min, 37 °C). Following trypsinization, floating and adhered cells were mixed and centrifuged (400× *g* for 5 min). The resulting cell pellets were resuspended in 100 μL of PBS and treated with 4.5 μL/sample of Annexin V Conjugates for Apoptosis Detection (BD Bioscience, Warsaw, Poland) with 8 µL/sample of propidium iodide (PI; BD Bioscience, Warsaw, Poland). After incubation for 15 min at 4 °C, cells were immediately analyzed using a flow cytometer (FACS Calibur; BD, San Jose, CA, USA). Acquisition was stopped after 8000 cells. The percentages of live, apoptotic, and necrotic cells were determined with CellQuest Pro Software (BD, San Jose, CA, USA) and presented as mean ± SD of three independent experiments (*n* = 9).

- Proliferation (Ki-67 assay) and cell cycle analysis

After trypsinization (0.05% trypsin, 3–7 min, 37 °C), cells were seeded in a 24-cell plate at a density of 2 × 10^4^ cells/well. After 24 h, cells were washed twice with a PBS solution. Then, fresh culture medium was added and peptides **1**–**3** at final concentrations of 0 (control), 0.01, 0.05, 0.1, 0.5, or 1 mM were applied. After 24 or 72 h, cells were collected (0.25% trypsin, 5–7 min, 37 °C) and washed twice with PBS. Subsequently, cells were resuspended in 1 mL of cold 70% ethanol/PBS solution (4 °C) and stored in a freezer (−20 °C) for 1−30 days. On the day of the analysis, cells were centrifuged (300× *g*, 5 min) and stained with Ki-67 as per the manufacturer’s instructions. PI staining was also performed as previously described [73,74]. For Ki-67 staining, 200 µL of PBS with 10 µL of mouse anti-Ki67 antibody (BD Bioscience, Warsaw, Poland) was added for 30 min at room temperature. For PI staining, 300 μL of stain buffer (PI/RNase Staining Buffer; BD Bioscience, Warsaw, Poland) and 10 μL of PI (BD Bioscience, Warsaw, Poland) were added for 15 min at room temperature. In both Ki-67 and cell cycle assays, cells were washed twice with a PBS solution after staining. Both assays were performed on a flow cytometer (FACS Calibur; BD, San Jose, CA, USA). For the proliferation (Ki-67) assay, the cell cycle analysis was stopped after 10,000 cells, and acquisition was stopped after 25,000 cells, as calculated using the FCS Express program (De Novo Software, Pasadena, CA, USA). The results are presented as mean ± SD from three independent experiments (*n* = 9).

- Statistical analysis of studies on peptides 1–3

All results are presented as mean ± SD. The results were statistically analyzed using *t*-tests and one-way ANOVA with Bonferroni correction (in the case of normal distribution) or nonparametric Kruskal–Wallis and Mann–Whitney U tests (in the case of nonparametric distribution). Data distribution was evaluated using the Shapiro–Wilk test. GraphPad Prism software (version 7; GraphPad Software, Inc., La Jolla, CA, USA) was used for the statistical analysis. *p* < 0.05 was considered statistically significant for all analyses.

## 3. Results

### 3.1. Preparation and Characterization of Polysaccharide–Peptide Conjugates

Peptides H-RGDS-OH (**1**) and H-GRGDS-NH_2_ (**2**), as well as the linear precursor of cyclo(RGDfC) (**3**), were synthesized on solid phase using DMT/NMM/TosO^−^ as a coupling agent [65]. Cyclization was performed using the standard high-solution-dilution protocol [64]. The characteristics of the synthesized peptides **1**–**3** are presented in Table 3.

Using peptides **1**–**3** as components, peptide–polysaccharide conjugates were prepared, in which the aforementioned peptides were bound to solid substrates through a network of weak bonds (dip-coating method). The possible weak interactions determining the binding of peptides **1**–**3** with polysaccharides **A**–**C** are shown in Figure S9 in the Supporting Material. In order to obtain the conjugates of **A**–**C** polysaccharides with peptides **1**–**3**, the same amount of peptide was added to the nonwoven material (25 mg of the peptide was added to 25 cm^2^ samples of the nonwovens), which resulted in polysaccharide–peptide conjugates with a peptide concentration of 1 mg/cm^2^ of the polysaccharide matrix.

**Figure 1:**
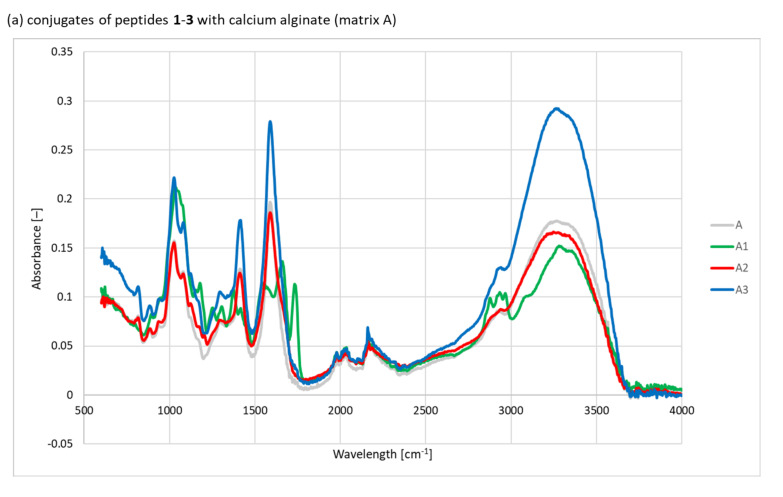

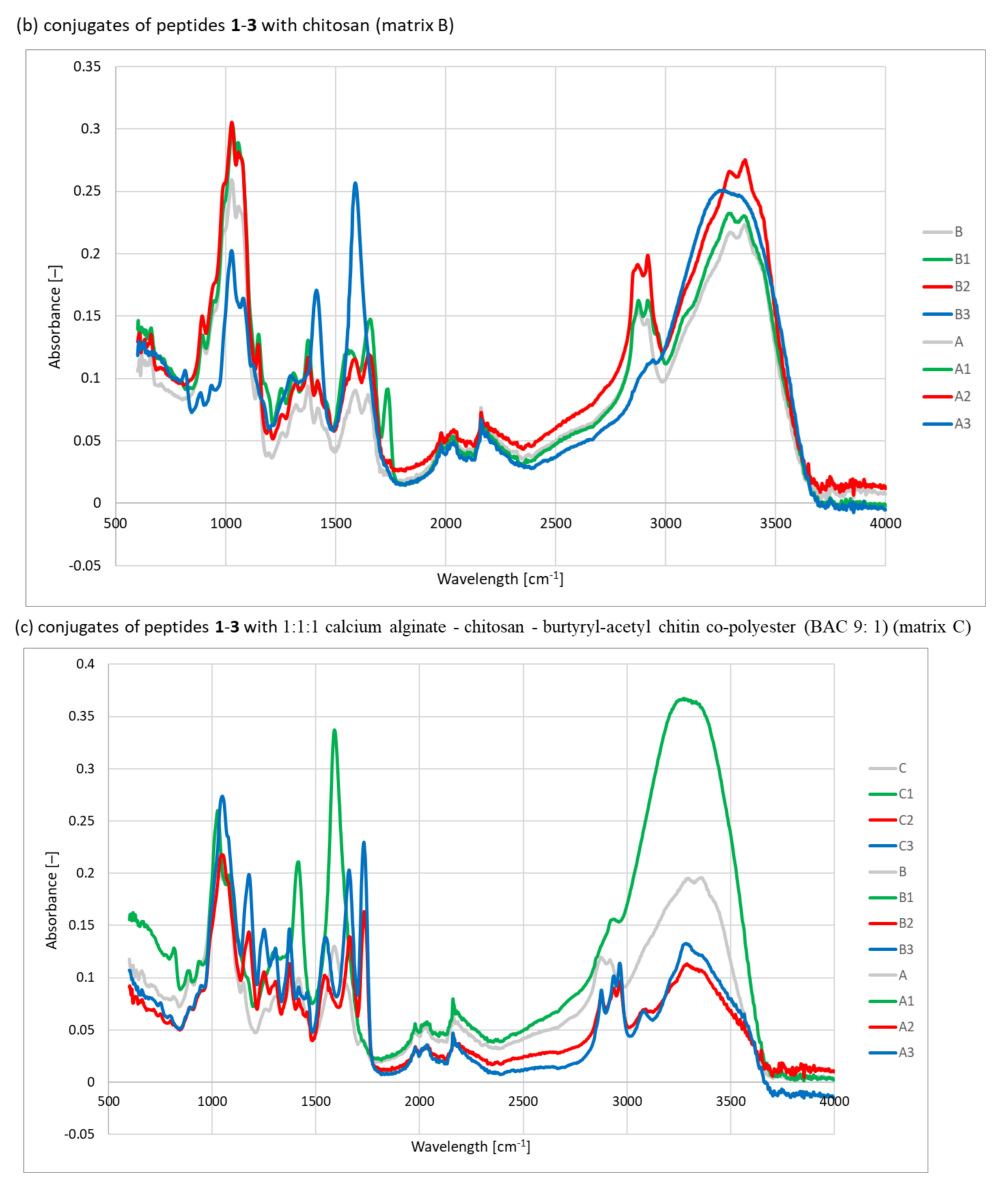
FTIR spectra of polysaccharide nonwovens **A**–**C** modified with peptides **1**–**3**.

The FTIR analysis confirmed the presence of peptides **1**–**3** and polysaccharides **A**–**C** in the final conjugates (Figure 1).

Spectral analysis revealed the presence of bands characteristic of peptide bonds at 1650 and 1550 cm^−1^. In addition, a band characteristic of peptide chains was found at 1300 cm^−1^. Unfortunately, other bands’ characteristics of peptides/proteins in the spectral range of 3500–3200 cm^−1^ were poorly visible, which is due to the presence of intense saccharide bands. Another band characteristic of peptides was found in the range of 1583–1484 cm^−1^, corresponding to the amide bond (amide II) derived from peptides **1** and **3**. Furthermore, bands were seen at 1718–1584 cm^−1^ (amide I) and at 1484–1583 cm^−1^ (amide II) for peptide **2**, confirming the modification of the polysaccharide matrices. The spectra for the conjugates of peptides with calcium alginate (matrix **A**) showed characteristic wide bands in the vibration range of 3500–3100 cm^−1^, which are typical of the stretching vibrations of OH group blocks M and G. Two bands relating to the valence vibrations of the C–O bond of the carboxylate ion were also visible at 1420 cm^−1^ (asymmetrical) and 1620 cm^−1^ (symmetrical). The most intense band found at 1050 cm^−1^ corresponded to the skeletal vibrations of the glycosidic bond (C–O–C). The spectra of peptides **1**–**3** conjugates with chitosan (matrix **B**) showed bands from the OH group (3000–3750 cm^−1^), CH_2_ group (ν = 2930 cm^−1^), and CH (ν = 2875 cm^−1^), which corresponded to the vibration bands of the CH bond. This is in line with the presence of the vibration bands at ν = 1380 cm^−1^ and ν = 1428 cm^−1^, which are typical of methylene groups. Bands observed in the spectral range of 1680–1480 cm^−1^ corresponded to the vibrations of the carbonyl group derived from amide (CONH-R). The most intense band for chitosan seen in the range of 1160–1000 cm^−1^ corresponded to the vibrations of the C–O group from the glycosidic bond (C–O–C) of the chitosan chain. The spectra of the peptide **1**–**3** conjugates with the **C** matrix containing equal amounts of calcium alginate, chitosan, and chitin butyryl-acetyl co-polyester (BAC 90:10) showed signals corresponding to calcium alginate and chitosan groups, as well as bands characteristic of BAC 90:10. In the BAC 90:10 spectrum, signals found at 1740 and 1250 cm^−1^ corresponded to the C=O carbonyl groups. Higher absorption at 2990–2850 cm^−1^, as well as at 790 and 740 cm^−1^, was also characteristic of the increased content of aliphatic –CH_2_ and –CH_3_ groups from butyric and acetic residues. The presence of GlcNHAc units was confirmed by bands at 1650, 1565, and 1412 cm^−1^, which corresponded to the amide groups I, II, and III, respectively. In the BAC 90:10 spectrum, a band for –CH_3_ in the acetylamide groups was observed at 1376 cm^−1^. Furthermore, a band for the C–O–C band in the glucopyranose ring was seen at 1028 cm^−1^, and bands for the β(1 → 4) glycoside bridge at 1153 and 895 cm^−1^.

**Figure 2:**
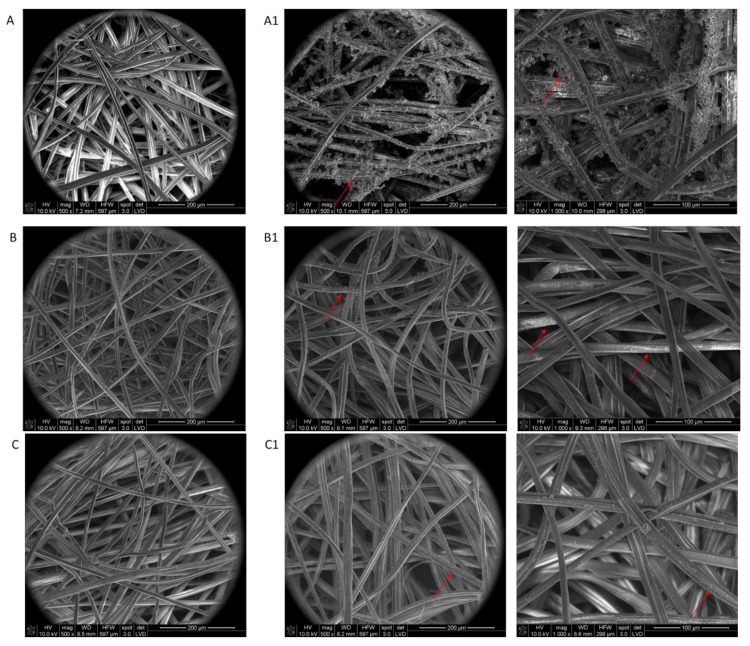
SEM images of unmodified nonwovens (**A**–**C**) and all polysaccharide nonwovens (**A1**–**C1**) modified with peptide **1** (magnification: 500× and 1000×). Arrows indicate positions on the fibers where peptide deposits are clearly visible.

To investigate whether the physical deposition (dip-coating method) of peptides **1**–**3** on polysaccharide nonwovens **A**–**C** had an influence on the material morphology by increasing the degree of fiber interaction, an SEM analysis was performed on unmodified nonwovens **A**–**C** and all polysaccharide nonwovens (**A1**–**C1**) (Figure 2). The effect of the dip-coating procedure on the morphology of the modified nonwovens was analyzed using peptide H-RGDS-OH (**1**).

The results of the SEM analysis indicated that the process of peptide deposition did not change the morphology of the studied fibers used for the preparation of nonwovens, and in the next step to modification with peptides, it can be seen that only the particle of the peptide is deposited on the unchanged fiber’s surface. The images showed no visibly tighter fiber packages (Figure 2) that can affect the ability of cells to interact with fibers or change the physicochemical and mechanical properties of nonwovens (Figure 2). In addition, there were no “sticky” areas in which the fibrous structure of the material was disturbed. The coating of the polysaccharide fibers varied depending on the material used. In the case of calcium alginate nonwoven (**A**) (Figure 2 (**A1**)), the cover layer formed by the deposition of peptide 1 did not adhere closely to the surface of the polysaccharide fiber (red arrow in Figure 2). This may be due to the partial repulsion of the carboxylate groups of peptide **1** and the carboxylate anions of the polysaccharide, causing a reduction in the stability of the conjugates. However, considering the release of the RGD peptide, this may be advantageous, as it can promote a faster release of the peptide. For chitosan (B) and ternary C nonwovens, the peptide layer adsorbed closely on the polysaccharide fiber (Figure 2 (**B2**,**C2**)).

### 3.2. Biological Activity of Polysaccharide–Peptide Conjugates Derived from Nonwovens **A**–**C** and Peptides **1**–**3**

**Figure 3:**
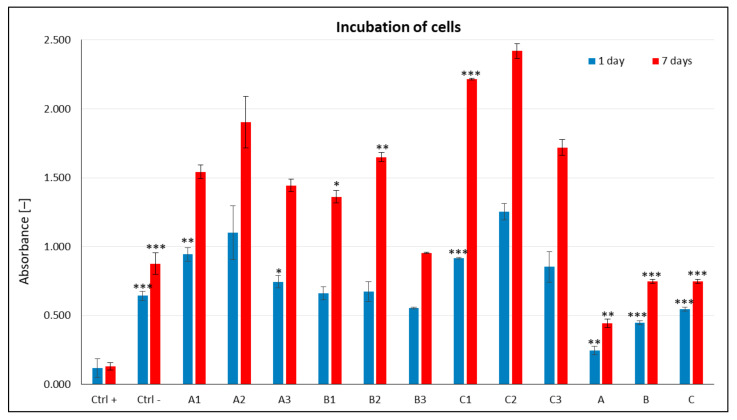
Metabolic activity of C2C12 cells cultured in the presence of peptide–polysaccharide conjugates. Cells were treated with the extracts of conjugates for 1 day (24 h) and 7 days (168 h). Cytotoxicity assessments were performed in five replicates (MTT test). Cells grown for 48 h in DMEM were used as a negative control, while those treated with dimethyl sulfoxide (DMSO, 5% of final concentration) were used as a positive control. All experiments were performed in triplicate. The results are expressed as a percentage of the negative control with the standard deviation (SD). Statistical significance was assessed using a one-way analysis of variance (ANOVA) and assumed at *** *p* < 0.001, ** *p* < 0.01, and * *p* < 0.05.

The effects of peptides **1**–**3** deposited on polysaccharide matrices **A**–**C** (Figure 3) were studied using C2C12 cells. Experiments were performed after a day or week of incubation to assess the short- and long-term effects of the peptide–polysaccharide conjugates, which is particularly important for the application of these materials in regenerative medicine. An MTT assay was carried out to determine the cytotoxicity of peptide–polysaccharide conjugates. Cells cultured without additives in the medium were used as a negative control (K^−^), while those grown in the presence of DMSO (5%) added on the second day of incubation served as a positive control (K^+^). Unmodified polysaccharide nonwovens were also studied in the experiments.

The results of the cytotoxicity study revealed that none of the tested peptide–polysaccharide conjugates induced cytotoxicity in C2C12 cells. In all cases, the absorbance values were significantly higher than or comparable to that of the control. After 24 h of incubation, the smallest positive effect on C2C12 cells was observed upon their incubation with conjugates containing chitosan matrix **B** (Figure 3). The data obtained for unmodified **A**–**C** nonwovens are statistically significantly lower than the absorbance values for extracts of peptide-modified **1**–**3** nonwoven polysaccharide nonwovens **A**–**C**. For conjugates with chitosan matrix, a more positive effect of peptides **1** and **2** was observed in the cells in comparison to cyclic peptide **3**. Significantly higher absorbance values were obtained for the conjugates containing the calcium alginate matrix (**A**) and the mixed polysaccharide matrix (**C**) containing equal amounts of calcium alginate, chitosan, and BAC 90:10, which suggests that these polysaccharides may be more useful for muscle tissue regeneration. Among the tested conjugates, the highest absorbance values were obtained for **A2** and **C2**, which indicates the positive effect of peptide **2** (Figure 3). No reduction in cell viability was observed after 72 h of incubation of the cells with the tested conjugates (Figure 3). Most importantly, the absorbance values increased over time, proving that peptide–polysaccharide conjugates can have potential applications in regenerative medicine. The chitosan (**B**) matrix showed the least positive characteristics among the polysaccharide matrices. Although significantly higher absorbance values were obtained for the conjugates with matrices **A** and **C**, better parameters were found for nonwoven C with equal amounts of calcium alginate, chitosan, and the relatively hydrophobic chitin butyryl-acetyl co-polyester (BAC 90:10). Peptide **2** was also found to have a positive effect on C2C12 cells, and all conjugates containing it showed higher absorbance values compared to the conjugates of peptides **1** and **3**. Peptide **1** had a positive effect on the viability of C2C12 cells with extended incubation.

**Figure 4:**
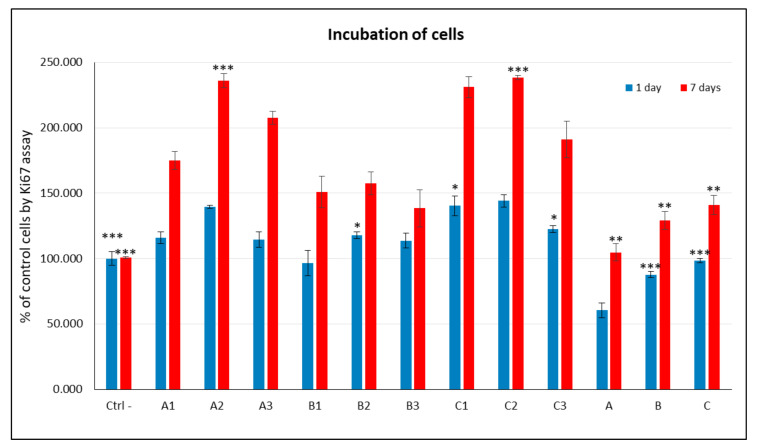
Assessment of the proliferation capacity of C2C12 cells cultured in the presence of extracts of peptide–polysaccharide conjugates **A1**–**A3**, **B1**–**B3**, **C1**–**C3** (% of control cells by Ki67 assay). Ki67 assay was performed on unmodified **A**–**C** polysaccharides and modified polysaccharides after 1 day (24 h) and 7 days (168 h) of incubation. Results are presented as mean ± SD from three independent experiments. Cells grown in DMEM without any additions was used as a negative control. Statistical significance was assessed using a one-way analysis of variance (ANOVA) and assumed at *** *p* < 0.001, ** *p* < 0.01, and * *p* < 0.05.

The proliferative capacity (Ki-67 assay) of C2C12 cells was analyzed following culture with extracts from peptide–polysaccharide conjugates **A1**–**A3**, **B1**–**B3**, and **C1**–**C3** (Figure 4). Ki-67 protein (MKI67) is a cellular proliferation marker, which is found during all active phases of the cell cycle (G1, S, G2, and mitosis), but not in the resting (quiescent) phase (G0) [75]. The cellular content of this protein markedly increases as cells progress through the S phase of the cell cycle [76]. In this study, the results of the Ki-67 assay showed that none of the extracts of the nonwovens inhibited the proliferative capacity of C2C12 cells. Peptides **1**–**3** deposited on the polysaccharide surface exhibited similar biological activity (Figure 4).

Regardless of the polysaccharide substrate used, H-GRGDS-NH_2_ (**2**) was found to be the most active. The lowest number of proliferating cells was observed for the group of nonwovens containing chitosan (**B**) as a solid substrate. On the other hand, for nonwovens **A** and **C**, a significantly higher percentage of proliferating cells was observed in comparison to both the control and the extracts of unmodified polysaccharide nonwovens (**A**–**C**).

**Figure 5:**
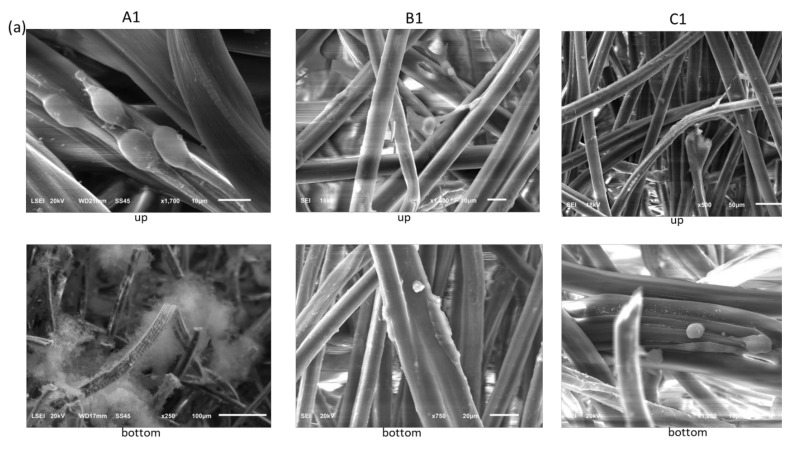

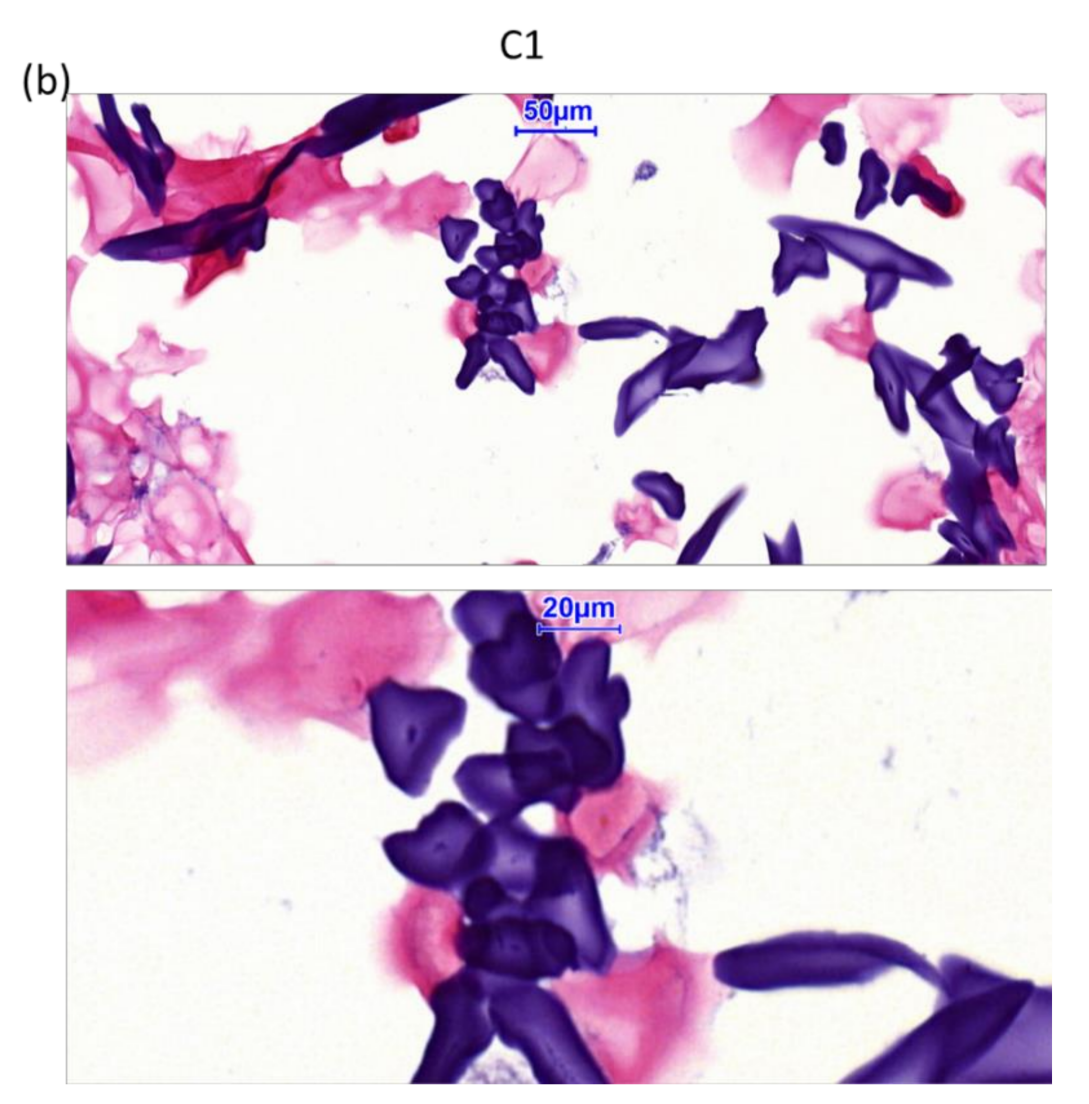
Microscopic evaluation of the growth of C2C12 cells on nonwovens **A1**, **B1**, and **C1**. (**a**) SEM images. (**b**) Images from light microscopy with hematoxylin–eosin staining. For SEM imaging, samples were taken from the upper (up) and lower (bottom) quadrants of nonwovens folded to fit the cell culture inserts.

Microscopic analyses were performed to assess the proliferative capacity of C2C12 cells and their morphology. In all cases of nonwoven fibers, cells strongly adhered to the surface of the polysaccharide fibers (Figure 5a). In the case of nonwoven **B**, a three-dimensional network was observed, in which the cells were connecting individual fibers in the nonwoven. Elongation of cells suggests the initiation of myoblast-to-myocyte differentiation. In this study, apart from cells interacting with fibers, haze-like structures (**A1** bottom) were also observed, which may indicate the initial phase of ECM formation.

For material **C1**, the classic staining procedure was used. Hematoxylin and eosin fibers were frozen at −20 °C and applied after 24 h of incubation (Figure 5b). Stained cells showed proliferative ability on the nonwoven. Nonwoven **C** was used for this analysis because it contained equal amounts of calcium alginate–chitosan and the BAC derivative, which represents a combination of acid, alkaline, and neutral fibers (not dyed by hematoxylin and eosin).

#### 3.2.1. Antimicrobial Activity of Polysaccharide–Peptide Conjugates

The antibacterial activity of the peptide–polysaccharide conjugates was assessed with the aim of selecting conjugates with inherent antibacterial activity. Unmodified polysaccharides **A**–**C** were used as controls for this analysis. According to the ISO 20743:2013 standard, a material with antibacterial activity (A) in the range of 2–3 is characterized as exhibiting significant antibacterial activity, while those with A > 3 are classified as highly antibacterial. The criteria applied for the assessment of antibacterial activity were as follows: (a) the concentration of inoculum ranged from 1 × 10^5^ to 3 × 10^5^; (b) the maximum difference between the three controls tested immediately after inoculation was ≤1; (c) for the control sample, the increase in the value of F was ≥1. The values of antibacterial activity determined for the tested materials are summarized in Table 4 and Table 5.

The highest value of antimicrobial activity against *S. aureus* ATCC 6538 was observed for conjugates **B1** and **B2**, which can be classified as highly antibacterial (Table 4). This can be related to the use of chitosan (**B**), which after protonation, gives chitosan ammonium salts with documented antibacterial activity as a matrix [77]. It must be stated that the chitosan substrate was not treated with acids before the application of peptides, so the observed effect resulted from the partial protonation of the polysaccharide amino groups by the peptides. This assumption is consistent with the A values determined for conjugates **B1** and **B2**. In the case of conjugate **B1**, peptide H-RGDS-OH (**1**) contains two carboxyl groups (A = 3.87). Because peptide H-GRGDS-NH_2_ (**2**) has one free carboxyl group in the Asp side chain, its A value was found to be lower (3.01). For chitosan conjugate with cyclo(RGDfC) (**3**), which also contains one carboxyl group, the value of A decreased to 2.85. A similar relationship between the number of carboxyl groups in the peptide and the A value was observed for matrix **C**, which contained two-thirds of the amount of chitosan in matrix **B**. Conjugates of peptides **1**–**3** with calcium alginate (matrix **A**) did not show antibacterial activity against *S. aureus* ATCC 6538.

For all the conjugates with chitosan (**B1**–**B3**), the values of antibacterial activity against *K. pneumoniae* ATCC 4352 were >3. Therefore, these conjugates can be classified as highly antibacterial materials (Table 5). Conjugate **C1**, which contained peptide **1** with two carboxyl groups and reduced chitosan content in the polysaccharide matrix, was also found to be highly antibacterial. For conjugates **C2** and **C3**, a reduction in the A value was observed, but the values were >2. Therefore, these conjugates can be classified as materials with significant antibacterial activity. None of the conjugates of peptides **1**–**3** with calcium alginate (**A1**–**A3**) showed antibacterial activity against *K. pneumoniae* ATCC 4352.

#### 3.2.2. Release Study of Peptides **1**–**3** from **A1**–**A3**, **B1**–**B3**, and **C1**–**C3** Materials

The release of an active compound/ingredient (API) leads to the liberation of the active substance into the surrounding liquid environment. Studies on the release of API are carried out as part of qualitative research in the drug manufacturing process. The results of such studies often correlate well with the pharmacokinetic parameters determined in vivo (bioavailability), as well as with the pharmaceutical availability of active substances, which is expressed as a percentage of the declared dose of API released into the acceptor fluid over a specified time period (release factor, Q). An unmodified and modified release profile is typical of a drug (API) (Figure S10 in the Supporting Material).

**Figure 6:**
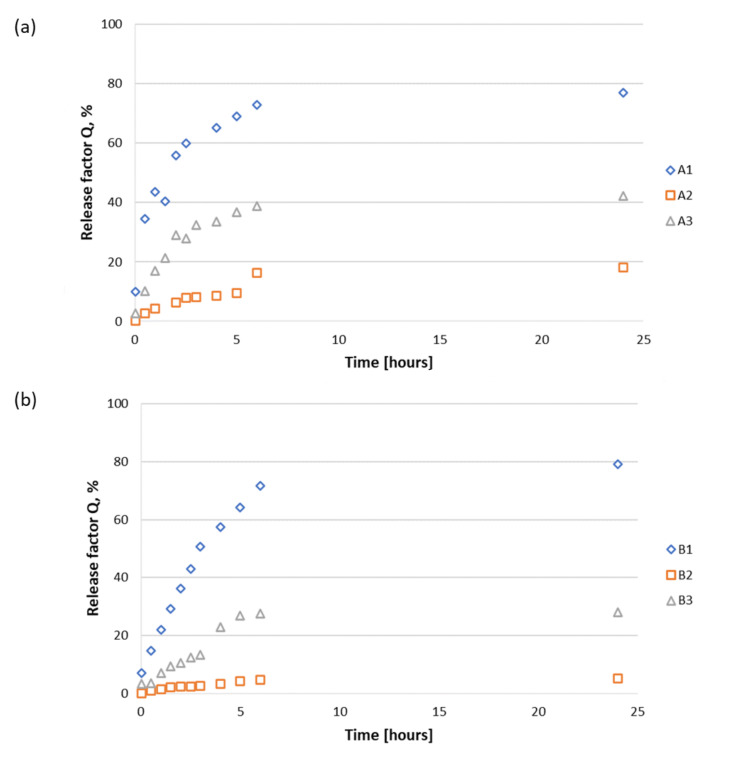

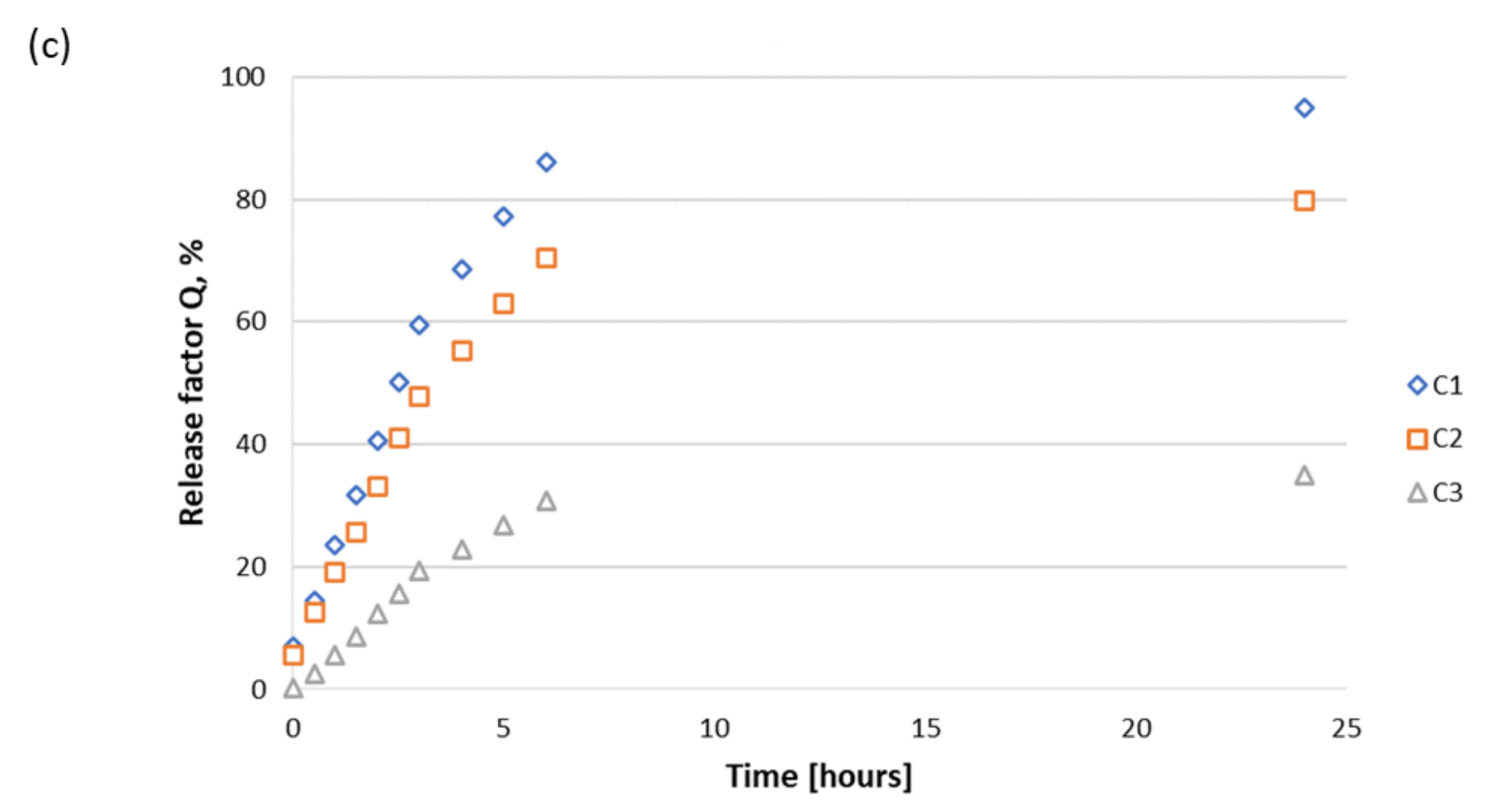
Release factor (Q) of RGD peptides **1**–**3** deposited on polysaccharide matrices **A**–**C**: (**a**) release of peptides **1**–**3** from the alginate matrix (**A**); (**b**) release of peptides **1**–**3** from chitosan matrix (**B**); (**c**) release of peptides **1**–**3** from a matrix composed of calcium alginate, chitosan, and chitin butyryl-acetyl co-polyester (BAC 9:1) in 1:1:1 ratio (**C**).

**Figure 7:**
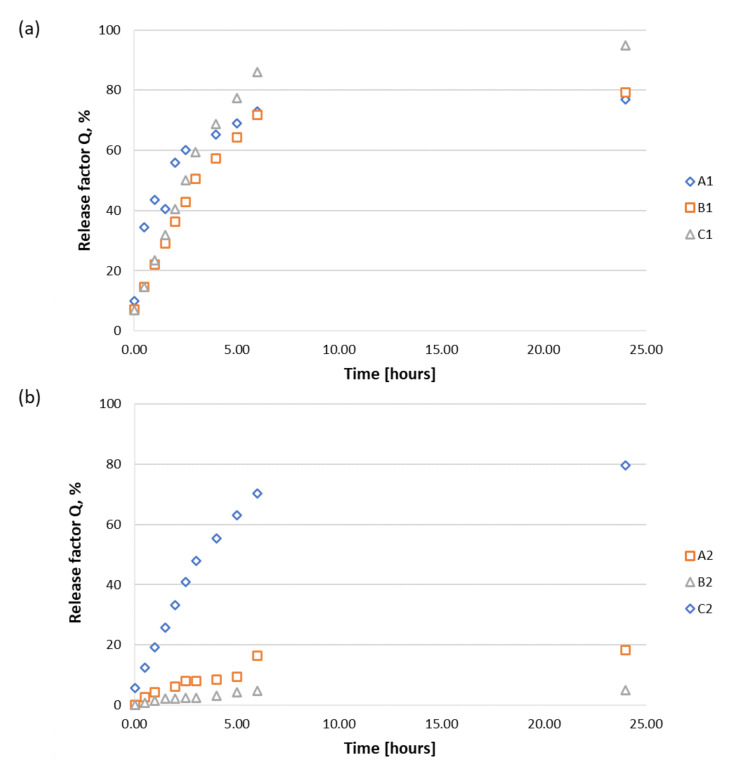

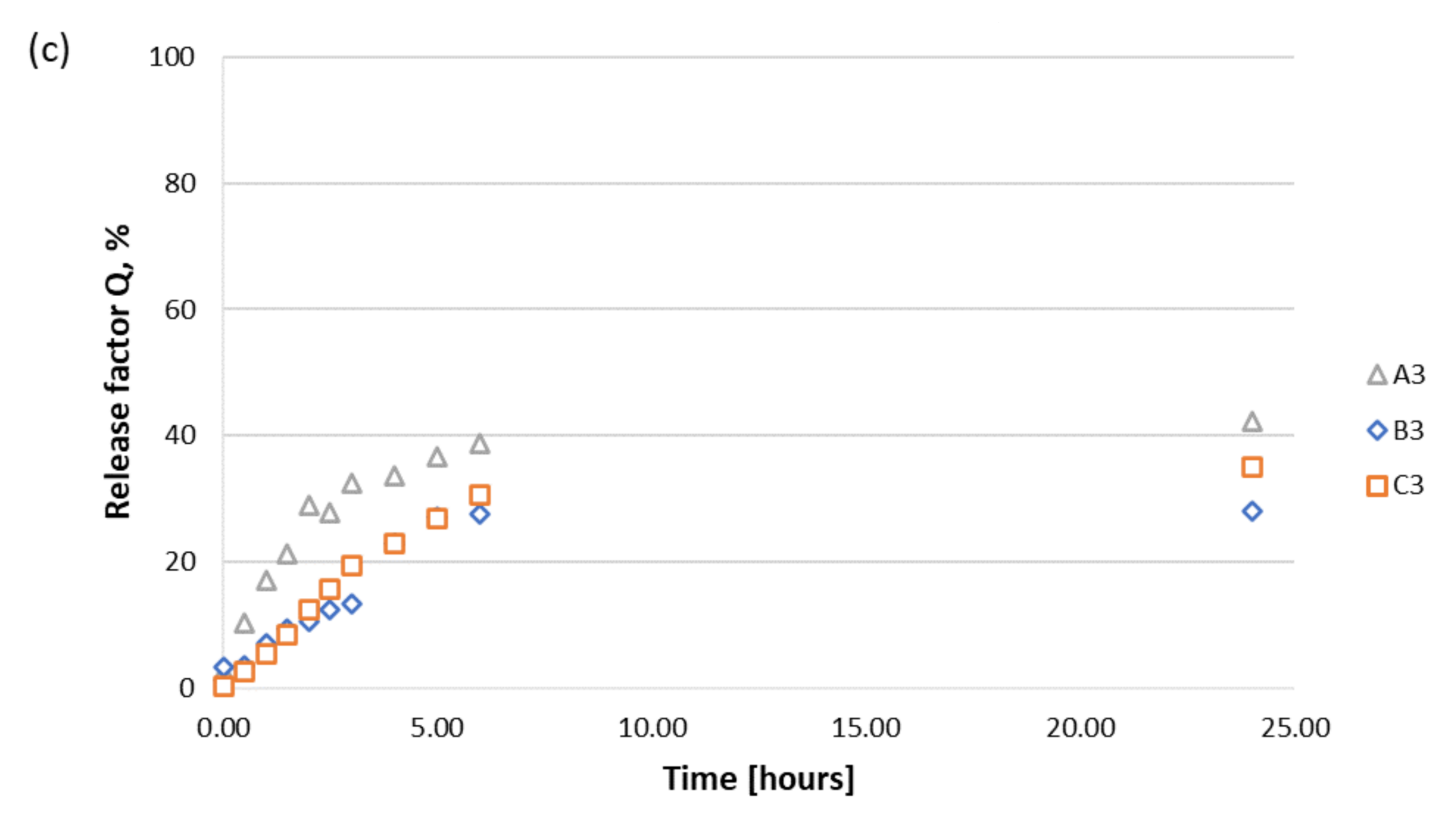
Release factors of RGD peptides **1**–**3** deposited on polysaccharide matrices **A**–**C**: (**a**) release of H-RGDS-OH (**1**) from matrices **A**–**C**; (**b**) release of H-GRGDS-NH_2_ (**2**) from matrices **A**–**C**; (**c**) release of cyclo(RGDfC) (**3**) from matrices **A**–**C**.

In this study, we investigated the ability of the RGD peptides (model APIs) to be released from the solid support, considering the weak bonds between peptides **1**–**3** and the polysaccharide matrices **A**–**C**. The percentage concentration of released active substances (release factor, Q) was determined based on the absorbance measurements (Figure 6 and Figure 7). All the polysaccharide–peptide materials were evaluated in release studies, and the materials used in peptides **1**–**3** were completely characterized (Table S3 in the Supporting Material). Phosphate buffer (pH 7.0, PBS 1× concentrated) was used as an acceptor liquid in the analysis. Peptide release was performed at 37 °C (Figure S11 in the Supporting Material). Calibration curves (Table S3 and Figure S12 in the Supporting Material) generated for peptides **1**–**3** were used to determine the release factor Q and the correlation equations for conjugates **A1**–**A3**, **B1**–**B3**, and **C1**–**C3**.

Analysis of various correlation equations (linear regression, exponential regression, logarithmic regression, power law regression) showed that it is impossible to find one type of correlation equation for all the tested materials (see Table S4 in the Supporting Material). This may be related to the differences in the properties of both peptides **1**–**3** and polysaccharides **A**–**C,** and individual consideration of each material has to be used to approximate the release process to the mathematical model.

For conjugates **A1**, **B1**, and **C1**, the initial burst of peptide **1** release at an amount of 10–30% occurred very quickly upon immersion of the conjugates into the acceptor fluid—immediately after 1 min for conjugate **A1** and from 1 min up to 0.5 h for conjugates **B1** and **C1**. The second release stage, in which 40–60% of peptide **1** was released, began after 0.5 h and occurred up to 2 h for conjugate **A1**, at 1 h up to 1.5 h for conjugate **B1**, and at 1.5 h up to 2 h for conjugate **C1**. Finally, the release of at least 75% of peptide **1** was observed after a couple of hours for **A1**, **B1**, and **C1** (>4, 2.5, and 2.5 h, respectively). These results indicate the analogous release profiles of H-RGDS-OH (**1**), regardless of the carrier on which peptide **1** was applied (Figure 7a). The release curve profiles (Figure 6a–c) and the correlation equations (Table S4) describing those curves showed fairly significant sorption of peptides **1**–**3** by the fibrous carrier on which they were deposited. This is due to the fact that all the carriers used were polysaccharide nonwovens with significant sorption properties. For conjugates **A2**, **B2**, and **C2**, the first burst of peptide release at an amount of 10–30% was noticed only for calcium alginate. For chitosan and chitin butyryl-acetyl co-polyester matrix (**C**), the initial burst of peptide release was observed at 0.5 h up to 1.5 h upon immersion into the acceptor fluid. Only for **C2**, the release of 40–70% of the peptide was noticed after an incubation period of 2.5–5 h upon immersion into the acceptor fluid. Release of at least 80% of the peptide was observed after 24 h for the **C2** sample, which is similar to the previous release stages mentioned above. Samples **A2** and **B2** released <20% of peptide **2** within 24 h. Nevertheless, their release curves (Figure 6a–c) and correlation equations (Tables S3 and S4) indicated the clearly marked sorption of peptide **2** by the fibrous carrier. Moreover, in the initial phase, the release of the peptide was rectilinear and most visible in the case of sample **B2** (Figure 6b). The strong sorption and hydrophilic nature of the carrier material disrupted the process of simultaneous equilibrium sorption and desorption of peptide **2**.

For conjugates **A3**, **B3**, and **C3** containing cyclo(RGDfC) peptide (**3**), the first burst of peptide release at an amount of 10–30% was noted up to 1.5 h, at 2 h up to 24 h, and at 2 h up to 5 h, upon immersion into the acceptor fluid, respectively. The second release stage, with peptide release of 40–60%, was observed at 5 h up to 24 h for conjugates **A3**, while for samples **B3** and **C3**, the release factor did not exceed 37% even after 24 h of immersion into the acceptor fluid. The analysis of release curves (Figure 6a–c) and correlation equations (Tables S3 and S4) indicated the clearly marked sorption of the peptide by the fibrous carrier.

The highest degree of release from the alginate matrix (matrix **A**) (Figure 6a) was found for H-RGDS-OH (**1**), with a release factor (Q) value of 75% after 6 h of incubation. A slight increase in Q up to 80% was found after 24 h. A significantly lower release ability (Q ~ 40% after 24 h of incubation) was found for cyclo(RGDfC) (**3**). The lowest value of Q (20%) was found for H-GRGDS-NH_2_ (**2**). H-RGDS-OH (**1**) was very fast and effectively released from chitosan matrix **B** (Figure 6b) and ternary nonwoven **C** (Figure 7c). The high Q values for peptide **1** containing two free carboxyl groups indicate a possibility of partial protonation of the chitosan fibers, forming soluble chitosan salts. The release of peptides 2 and **3** from chitosan material **B** was inefficient, with Q values not exceeding 5% and 25%, respectively. On the other hand, the release of peptides **2** and **3** from matrix **C (**Figure 6c) was significantly higher, with a Q value of 80% and approximately 38%, respectively, after 24 h of incubation.

These results indicate that the structure of the peptide deposited on the nonwovens also influences the rate and efficiency of release. The used peptides differ in the number of free carboxyl groups, which form sodium salts in the presence of a phosphate buffer at pH 7.0, increasing the susceptibility of the peptide to release into the medium. Peptide **1** has two carboxyl groups, and peptide **2** has a free group on the aspartic acid side chain. Despite the presence of an aspartic acid residue, peptide **3** is significantly more hydrophobic than peptides **1** and **2**. The properties of peptides **1**–**3**, as described by Lipinski’s rules, are presented in Table 6.

We also evaluated the influence of the nonwoven structure of polysaccharides on the release profiles of peptides **1**–**3** (Figure 7). It was observed that for both chitosan matrix **B** and the ternary nonwoven **C**, ~80% of the H-RGDS-OH peptide (**1**) was released after 5 h (Figure 7b,c). Calcium alginate nonwoven **A** also allowed for the effective release of peptide **1**, but the process was significantly slower (Q = 78% after 24 h of incubation).

The most efficient release of H-GRGDS-NH_2_ (**2**) was observed for the conjugate with nonwoven matrix **C**, for which the Q value was determined at about 80% after 24 h of incubation (Figure 7b). On the other hand, the release efficiency of peptide **2** from conjugates **A2** and **B2** was significantly lower. For the conjugate with calcium alginate (**A**), the value of Q was 20% after 24 h of incubation, and for chitosan matrix **B**, it was only about 5%. Different results were observed for peptides **1**–**3** deposited on chitosan matrix **B**. The highest release rate was observed for the most hydrophobic peptide, cyclo(RGDfC) (**3**). After 24 h of incubation, the rate of release of the peptide from conjugate **A3** with calcium alginate was ~40% (Figure 7c), while for the remaining conjugates of peptide **3** with chitosan **B** and mixed nonwoven **C**, it was comparable at approximately 25% and 35%, respectively.

Although no strong bonds, such as covalent bonds, were observed between the peptides (API) and the fibrous polysaccharide carrier (different types of nonwovens), we achieved an extended-release dosage that could provide the appropriate therapeutic effect. Release studies demonstrated that the release profiles of all the tested conjugates (**A1**–**A3**, **B1**–**B3**, **C1**–**C3**) were typical of extended-release dosage forms. In all cases, the release of API did not exceed 65% of the total dose after 20–30 min of incubation, which can be related to the use of polysaccharide matrices as carriers. In an aqueous environment, polysaccharide matrices form a high-viscosity gel that not only hinders the diffusion of peptides into the fluid but also influences the sorption of API by the fibrous carrier. Despite the fact that peptides have a short diffusion path (from the surface of the fibers) and a gel-like structure, the strong sorption and hydrophilic nature of the carrier material disrupted the simultaneous equilibrium sorption and desorption of API.

### 3.3. Biological Activity of Peptides **1**–**3**

#### 3.3.1. Influence of Peptides **1**–**3** on the Viability of C2C12 Cells

**Figure 8:**
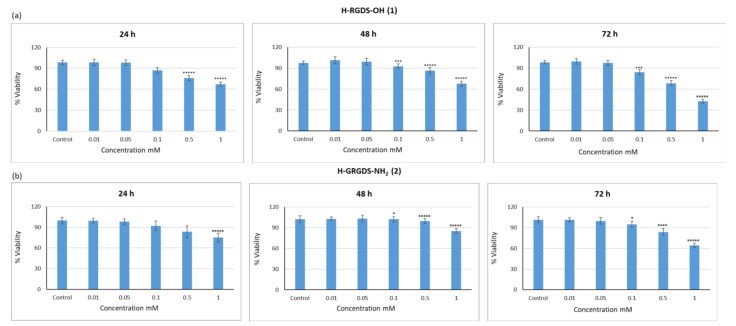

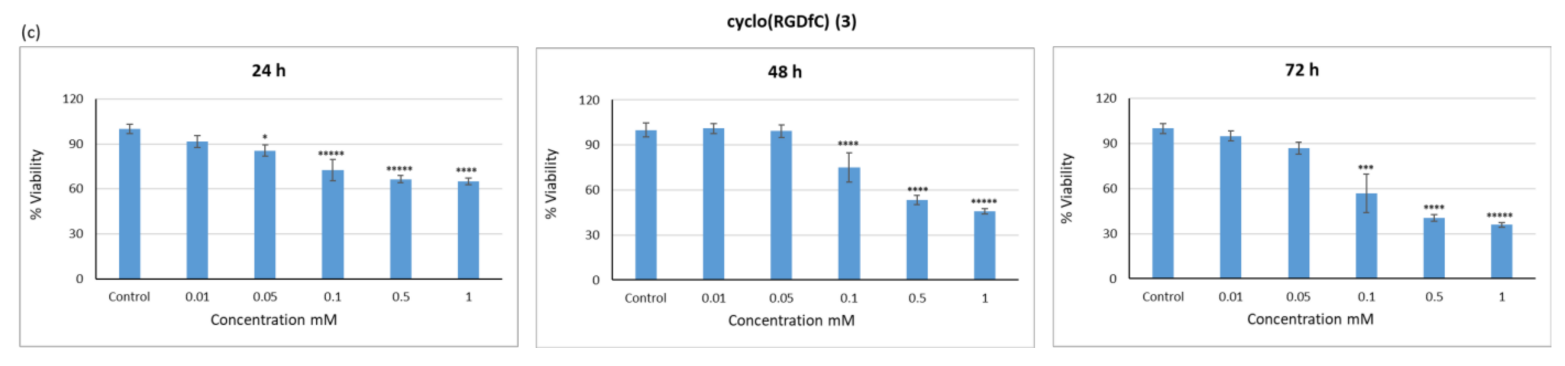
Cell viability of C2C12 cells cultured in the presence of peptides **1**–**3** was measured by Alamarblue^®^ assay after 24, 48, and 72 h, (**a**) results of tests in the presence of peptide **1**, (**b**) results of tests in the presence of peptide **2**, (**c**) results of tests in the presence of peptide **3**. Results are presented as mean ± SD of three independent experiments (*n* = 12). * *p* < 0.05; ** *p* < 0.01; *** *p* < 0.001; **** *p* < 0.001; ***** *p* < 0.0001.

The results from the analysis of the effect of polysaccharide conjugates with peptides containing the RGD motif on cell viability encouraged us to perform in-depth studies on the effects of peptides **1**–**3** on the C2C12 cells. Such an assessment of cell viability can indirectly reveal the damages to the cell membrane caused by the release of LDH. Resazurin-based assays are useful for quantifying the metabolic activity of viable cells. Resazurin (7-hydroxy-3H-phenoxazin-3-one-10-oxide) is a non-fluorescent blue dye that reduces to resorufin, a highly fluorescent pink dye, in the presence of metabolically active cells [78]. Damaged and nonviable cells cannot maintain the high reducing power, resulting in a proportionally lower resorufin signal. In this study, the cell viability of C2C12 cells was assessed using Alamarblue^®^ and LDH assays (Figure 8 and Figure S13 in the Supporting Material).

For the Alamarblue^®^ assay, peptides **1**–**3** were applied at varying concentrations, namely 0.01, 0.05, 0.1, 0.5, and 1 mM. Samples were compared to controls incubated without peptides (c = 0). In order to evaluate if the duration of incubation had any effect, the cells were analyzed at 24, 48, and 72 h. It was found that the application of H-RGDS-OH (**1**) at the highest concentration (c = 1 mM) caused a slight decrease in cell viability, which was observed after incubation for 24 and 48 h. Prolonged exposure to this peptide (72 h) resulted in a significant reduction in cell viability (35%), whereas at lower concentrations, peptide **1** did not have any effect (Figure 8a). Similar results were observed for H-GRGDS-NH_2_ (**2**) (Figure 8b), but the peptide concentration and the incubation time had no influence on cell viability. After 72 h of incubation with peptide 2 at a concentration of 1 mM, the cell viability was determined at 65%. In the case of cyclo(RGDfC) (**3**), both peptide concentration and incubation time were found to be the greatest influencing factors of cell viability (Figure 8c). An acceptable level of cell viability was observed with peptide **3** at concentrations of 0.01 and 0.05 mM and for all incubation times. At higher concentrations of 0.1, 0.5, and 1 mM, peptide **3** caused a decrease in cell viability, and this effect was most evident after 72 h of incubation, during which the cell viability was reduced to 57%, 40%, and 36%, respectively. Thus, the results of the Alamarblue^®^ assay, which evaluates the redox processes of metabolically active cells, suggested that cyclic peptide **3** may have limited application potential in regenerative medicine as it requires materials that can be safely used over long periods of time.

To confirm this observation, an alternative method, LDH assay, was used to assess cellular viability. This assay measures cytotoxicity by quantifying the activity of cytoplasmic enzymes released by damaged cells. When the cell membrane is damaged, LDH is rapidly released into the supernatant. Measuring the reduction of a yellow tetrazolium salt INT by NADH to a red, water-soluble formazan (with absorbance measured at 492 nm) enables the quantification of LDH activity. The amount of LDH released is proportional to the number of dead or damaged cells. In this study, no increase in the cellular level of LDH was caused by peptides **1**–**3** at any of the tested concentrations after incubation for 24 h. This indicates that peptides 1–3 had no cytotoxic effect (Figure S13). Interestingly, a slight decrease in LDH level was found in the samples incubated with low concentrations of peptides **1**–**3** (c = 0.01, 0.05, and 0.1 mM) in comparison to the control sample (not incubated with peptides). This indicates that the tested peptides had a positive effect on cell viability.

In the case of H-RGDS-OH (**1**) and cyclo(RGDfC) (**3**) peptides, extending the time of incubation of cells to 72h (Figure S13a–c) caused a significant increase in LDH levels. After incubation with peptide **3**, the level of LDH was more than doubled. By contrast, the H-GRGDS-NH_2_ (**2**) peptide did not have any adverse effect on cells during extended incubation (Figure S13b). At all peptide concentrations, the levels of LDH were slightly lower in the tested samples after 72 h of incubation in comparison to the control. In in vivo research investigating the use of materials as scaffolds, it is essential to ensure that the materials are nontoxic and to use more than one assay to determine their cytotoxicity. In this study, the highest (1 mM) peptide concentration was found to be toxic to cells. Among the tested peptides, the unfavorable cytotoxic effect of peptide **2** was the least significant.

#### 3.3.2. Genotoxicity of Peptides **1**–**3**

**Figure 9:**
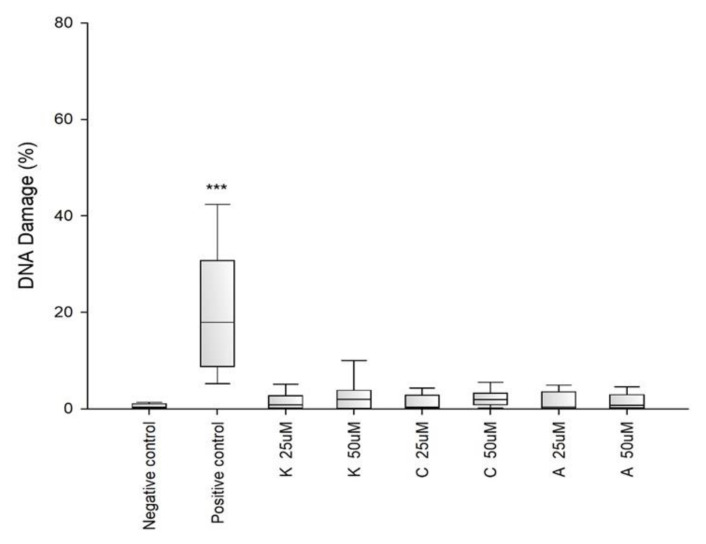
Genotoxicity of collagen peptides. Data were obtained from three independent tests. *** *p* < 0.001.

An alkaline version of the comet assay was used to assess the level of DNA damage caused by the tested variants of chitosan gels. This assay enables the quantification of oxidative damage, single- and double-stranded breaks, and alkaline labile sites. The amount of DNA damage is estimated from the percentage of DNA in the comet tail. In this study, the results of the applied comet assay showed that none of the tested peptides **1**–**3** at any concentrations induced significant DNA damage in C2C12 cells (Figure 9).

#### 3.3.3. Assessment of Cell Viability, Apoptosis, and Necrosis of C2C12 Cells Treated with Peptides **1**–**3**

**Figure 10:**
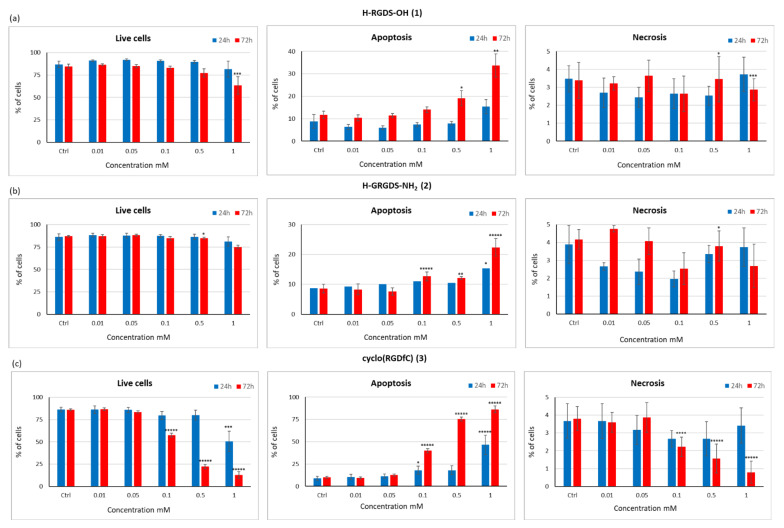
Cell viability, apoptosis, and necrosis of C2C12 cells cultured in the presence of peptides **1**–**3** were evaluated using Annexin V and PI assay after 24 and 72 h, (**a**) results of tests in the presence of peptide **1**, (**b**) results of tests in the presence of peptide **2**, (**c**) results of tests in the presence of peptide **3**. Results are presented as mean ± SD of three independent experiments (*n* = 9). * *p* < 0.05; ** *p* < 0.01; *** *p* < 0.001; **** *p* < 0.0001; ***** *p* < 0.00001.

The results of the LDH, Alamarblue^®^, and genotoxicity assays prompted us to investigate the possible mechanism by which these peptides influenced the viability of C2C12 cells. Three potential mechanisms were assumed: (1) induction of cell death (apoptosis/necrosis); (2) reduction of proliferation; and (3) cell cycle defects. First, the ratio of live, apoptotic, and necrotic cells was estimated in cultures treated with peptides **1**–**3** for 24 or 72 h (Figure 10). Then, the effects of peptides **1**–**3** on cell viability, apoptosis, and necrosis were evaluated by varying their concentrations (0.01, 0.05, 0.1, 0.5, and 1 mM).

In cultures treated with H-RGDS-OH (**1**) peptide, at all tested concentrations, the percentage of viable cells was comparable to that in the control after 24 h of incubation (Figure 10a). In cultures treated with peptide **1** at a concentration range of 0.01–0.5 mM, the proportion of apoptotic and necrotic cells was lower than that in the control. The only visible difference was observed in the case of culture treated with peptide **1** at a concentration of 1 mM, in which the share of apoptotic cells was almost doubled compared to that of control (8.7% vs. 15.4%). Incubation with H-GRGDS-NH_2_ (**2**) peptide caused no significant difference in cell survival at all tested concentrations (Figure 10b). At the highest concentration of peptide **2**, only an increase in the number of apoptotic cells was observed, but the proportion of necrotic cells remained unchanged. With 0.5 and 1 mM of peptide **2**, the proportion of necrotic cells was comparable to that of the control, while at lower concentrations, it was lower (Figure 10b). The highest concentration of cyclo(RGDfC) (**3**) caused a significant reduction in the proportion of viable cells, with the percentage of live cells reduced by 30% compared to the control (Figure 10c). Importantly, no increase in the proportion of necrotic cells was observed. In turn, a significant increase in the number of apoptotic cells (from 9% in the control to 46.6%) was observed.

In order to determine the effect of incubation time on cell viability, analogous tests were performed after 72 h of incubation (Figure 10). In the case of peptide **1**, after 72 h of incubation, a reduction in the number of viable cells was noted only at the highest concentration. The proportion of viable cells was 20% lower compared to that in the control. However, no increase in the number of necrotic cells was observed (Figure 10a). At a concentration of 1 mM, treatment with peptide **1** caused a significant increase in apoptotic cells (from 12% in the control to 34%). Similar results were obtained for H-GRGDS-NH_2_ (**2**) after 72 h of incubation. Even at the highest concentration of this peptide, only a negligible reduction in the number of viable cells was observed compared to the control sample (Figure 10b). Moreover, no increase in the number of necrotic cells was noted at any of the tested concentrations. At higher concentrations of peptide **2**, a slight increase in the number of apoptotic cells was observed. At the highest concentration of this peptide, the highest number of apoptotic cells (22%) was observed, which was about threefold higher than that in the control sample. In the case of the cyclic peptide (RGDfC) (**3**), extending the incubation time to 72 h resulted in significant cytotoxicity (Figure 10c). Only at the two lowest concentrations the cellular viability of the tested samples was comparable to that of the control. At 1 mM, peptide **3** caused a reduction in the number of viable cells to 13%, corresponding to an increase in the number of apoptotic cells, while apoptotic cells accounted for as much as 86% of all cells. However, the number of necrotic cells was small. It can be concluded that the cytotoxic effects of peptides **1**–**3** observed in Alamarblue^®^ and LDH assays were associated with the induction of apoptosis. Among the tested peptides, peptide **3** showed the highest cytotoxicity, especially after 72 h (Figure 10c).

#### 3.3.4. Influence of Peptides **1**–**3** on the Proliferation Capacity and Cell Cycle of the C2C12 Cell Line

**Figure 11:**
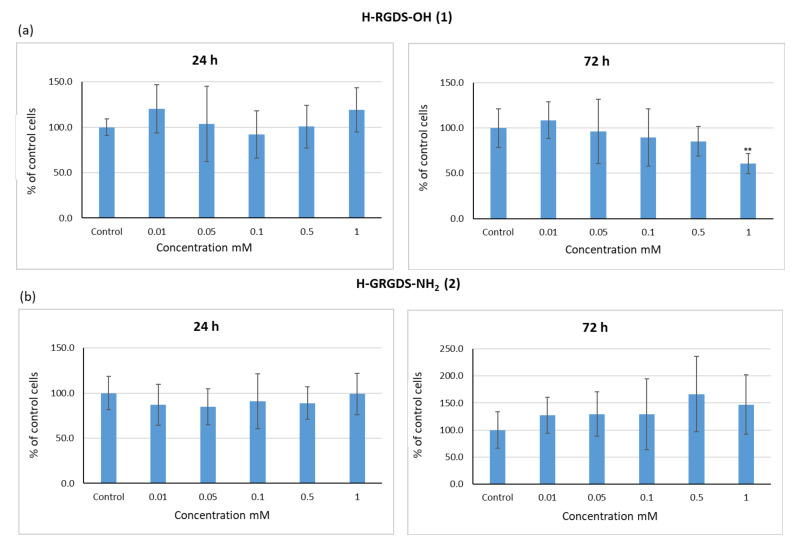

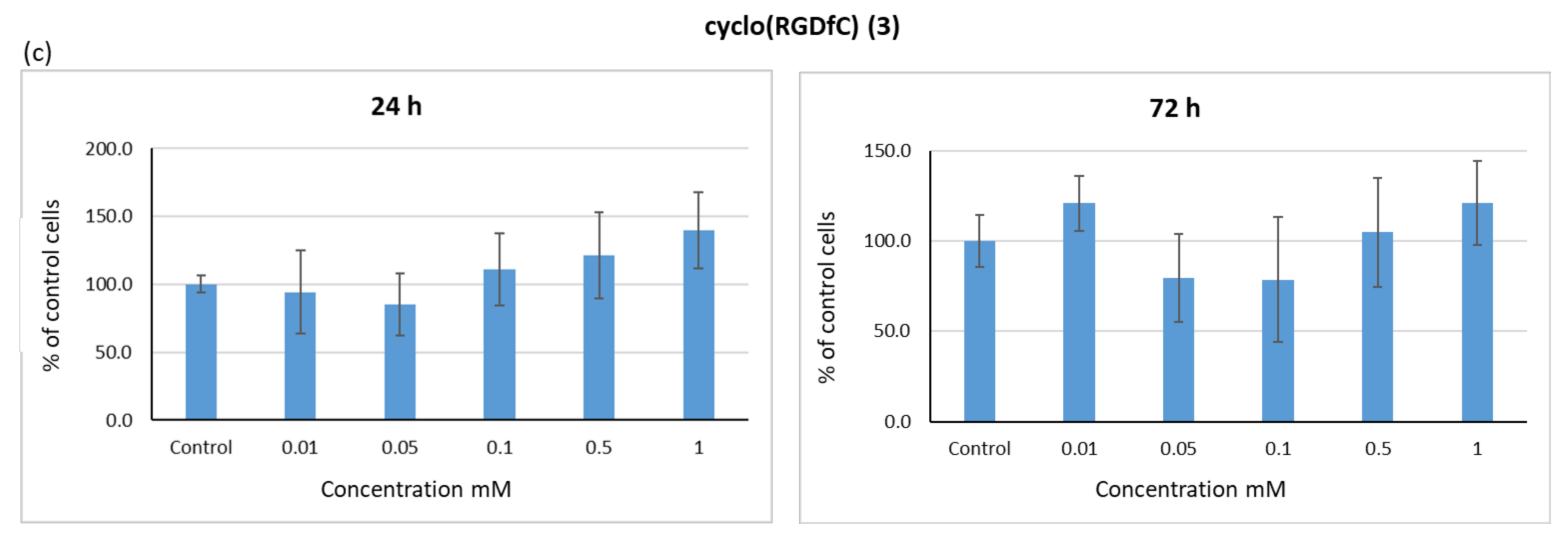
Proliferative capacity of C2C12 cells cultured in the presence of peptides **1**–**3** assessed using Ki-67 assay after 24 and 72 h, (**a**) results of tests in the presence of peptide **1**, (**b**) results of tests in the presence of peptide **2**, (**c**) results of tests in the presence of peptide **3**. Results are presented as mean ± SD from three independent experiments (*n* = 9), ** *p* < 0.01.

Cells should have the ability to regenerate and proliferate in the presence of biomaterials. In this study, we determined the proliferative capacity (Ki-67 assay) of C2C12 cells cultured in the presence of peptides **1**–**3** to test the ability of these peptides to support cell regeneration and proliferation (Figure 11).

Following 24 h of culture with H-RGDS-OH (**1**), the proliferative capacity of cells was comparable to, or even higher than, that of the control (Figure 11a). Extending the incubation time to 72 h caused a reduction in the proliferative ability of cells. This effect was proportional to the concentration of peptide **1**. At the highest concentration (1 mM), the proliferative capacity of cells decreased by 40%. Similar to the findings observed for peptide **1**, the proliferative ability of cells incubated with peptide H-GRGDS-NH_2_ (**2**) for 24 h was comparable to that of the control, regardless of the peptide concentration (Figure 11b). However, the most positive effect was observed when the incubation time was extended. The highest level of proliferating cells (160%) was observed in cultures treated with peptide **2** at a concentration of 0.5 mM. This may be related to the lowest level of apoptosis. In turn, regardless of the incubation time, cyclic peptide (RGDfC) (**3**) had a neutral or positive effect on cell proliferative ability at almost all of the tested concentrations (Figure 11c).

**Figure 12:**
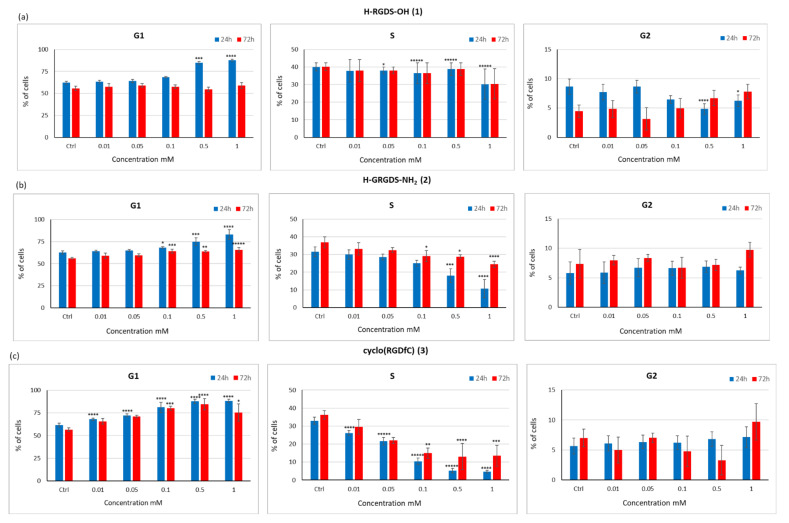
The cell cycle of C2C12 cells cultured in the presence of peptides **1**–**3** was assessed using PI assay after 24 and 72 h, (**a**) results of tests in the presence of peptide **1**, (**b**) results of tests in the presence of peptide **2**, (**c**) results of tests in the presence of peptide **3**. Results are presented as mean ± SD from three independent experiments (*n* = 9). * *p* < 0.05; ** *p* < 0.01; *** *p* < 0.001; **** *p* < 0.0001; ***** *p* < 0.00001.

The effect of peptides **1**–**3** on the cell cycle was assessed after 24 and 72 h of incubation (Figure 12). We evaluated the cell distribution across G1, S, and G2 cell cycle phases following treatment with peptides **1**–**3**. In the cell cycle, the G1 phase mainly involves the synthesis of enzymes that are needed for DNA replication in the S phase. The G2 phase involves the synthesis of proteins (mainly tubulin) that are needed for the formation of microtubules, a spindle component necessary for mitosis. Inhibition of protein synthesis in this phase prevents the cells from entering mitosis. In this study, samples cultured for 24 h in the presence of peptide **1** showed a more than 20% increase in G1 cells at the highest peptide concentration (Figure 12a). On the other hand, no such effect was observed for the concentrations of 0.01–0.1 mM, at which the number of cells in the G1 phase was comparable to that in the control. The proportion of cells in the S phase was comparable to that in the control cultures in the presence of peptide **1**, and only at the highest concentration, a 10% reduction in the number of cells was observed (Figure 12a). Analysis of cells in the G2 phase indicated that peptide 1 had a neutral effect on the synthesis of proteins needed for the M phase.

When H-GRGDS-NH_2_ (**2**) was used in the culture medium, an increase in the proportion of cells in the G1 phase was observed at 24 h. This increase was proportional to the concentration of the peptide used (Figure 12b). At the highest concentration, a 20% increase in the proportion of cells in the G1 phase was observed compared to the control. By contrast, with increased concentrations of peptide **2**, a decrease in the proportion of cells in the S phase (from 30% to 10% for 1 mM) was observed. This suggests that inhibition of DNA synthesis may have an adverse effect on the regenerative process. However, accelerated cell division, especially in the long term, may also have a negative effect as it can induce cancer formation. In this study, the proportion of cells in the G2 phase in cultures containing peptide **2** was comparable to that of the control after 24 h (Figure 12b).

Similar results were observed for cyclo(RGDfC) (**3**) (Figure 12c). As the concentration of this peptide increased, the proportion of cells in the G1 phase also increased. In samples cultured in the presence of this peptide at concentrations of 0.5 and 1 mM, there was a significant decrease in the proportion of cells in the S phase to around 5% compared to 33% in the control.

In order to study the effect of peptides **1**–**3** on cell division, a cell cycle analysis was performed after 72 h of incubation (Figure 12). It was found that there was no relationship between the concentration of peptide 1 and the number of cells in the G1 phase (Figure 12a). In all cases, the number of G1-phase cells was estimated at approximately 50%. Similar results were observed for the proportion of cells in the S phase, with 0.001–0.5 mM of peptide **1**. The mean number of S-phase cells was approximately 40%, and only at the highest concentration (1 mM) a 10% decrease was observed. The concentration of peptide **2** had the most visible effect on the progression of cells to the G2 phase. At higher concentrations (0.1–1 mM), there was an almost linear increase in the proportion of cells in this phase. After incubation with peptide **2**, a slow increase in the number of cells in the G1 phase was observed as the concentration increased (Figure 12b). A contrasting relationship was observed for cells in the S phase, the number of which decreased with increasing concentrations of peptide **2**. The number of cells in the G2 phase remained at 7–10%, regardless of the concentration of peptide 2 used. In the case of peptide **3**, its concentration was found to have a considerable effect on the number of cells in the G1 and S phases (Figure 12c). With increasing concentration of this peptide in the range of 0.01–0.5 mM, an increase in the proportion of cells in the G1 phase was observed. However, an inverse relationship was found for the cells in the S phase, the number of which decreased from 33% to 24% with an increasing concentration of peptide **3**. The greatest proportion of cells in the G2 phase was found following incubation for 72 h in the presence of 1 mM of peptide 3. The increase in G1 cells and decrease in S cells could be associated with two processes: blocking of the G1 checkpoint or induction of apoptosis. G1 checkpoint is the primary control mechanism that determines whether or not a cell will divide. It is important to analyze several biological signals both from within and outside the cell, such as cell size, nutrient availability, stimulation by growth factors, and DNA integrity. If cells do not pass through the G1 checkpoint, they may leave the cycle and remain in the G0 “resting” phase or they may undergo apoptosis. Induction of apoptosis causes the inhibition of protein and DNA synthesis, which in turn contributes to an increase in the percentage of G1-phase cells. In this study, we observed no genotoxicity at any of the tested peptide concentrations. However, more research is needed to determine whether the changes observed in the cell cycle were caused by the G1 checkpoint or apoptosis.

## 4. Discussion

Peptides H-RGDS-OH (**1**), H-GRGDS-NH_2_ (**2**), and cyclo(RGDfC) (**3**) used in the study are known to influence the adhesion of cells to solid materials [79,80]. Among the tested peptides, H-RGDS-OH (**1**) is considered a synthetic cell adhesion factor, which can mediate the adhesion of ECM components to cells and take part in cellular interactions. This peptide has been shown to activate caspases 8 and 9. H-RGDS-OH also activates intracellular integrin-dependent tyrosine and serine–threonine kinases. This indicates that integrins, especially those containing αV and β1 subunits, act as RGD receptors that can increase the mRNA synthesis of TGF-β1 and thus its secretion. Furthermore, H-RGDS-OH increases the tyrosine kinase-dependent phosphorylation of HMC proteins and the activity of ILK [81]. Peptide **2**, H-GRGDS-NH_2_, has been shown to inhibit the adhesion of human ovarian carcinoma cells (OVCAR-3) to fibronectin but not their adhesion to laminin [82]. The peptide H-GRGDS-OH, which differs from peptide **2** only by the presence of a carboxyl group at the C-terminus, can bind osteopontin (OPN). Although native OPN is capable of binding cells, the product of OPN cleavage resulting from treatment with thrombin has a significantly greater cell-binding ability. H-GRGDS-OH also mimics the cellular binding site of many adhesive proteins in the ECM. In addition, this peptide causes the dissociation of alpha-actinin and vinculin from the focal contact sites [83,84]. Cyclo(RGDfC) (**3**) is an integrin avb3-affine peptide [85]. Due to their ability to interact with integrins, RGD peptides are often used as scaffold components to improve cell adhesion [55,56,57,59,60,61,62,63].

The choice of calcium alginate (matrix **A**) [86,87,88] and chitosan (matrix **B**) [89] as polysaccharide substrates was based on their proven usefulness in regenerative medicine. Despite its hydrophobic nature, butyryl-acetyl chitin co-polyester (BAC 9:1), a component of the ternary matrix **C**), has been found to promote tissue regeneration and wound healing [90,91,92]. A key feature of all the polysaccharides used in this study is their proven biodegradability in vivo (breakage of polysaccharide chains results in soluble forms that are easily removed by the body once the substrate has fulfilled its desired functions). Due to the presence of hydroxyl, carboxyl, and amino groups, polysaccharide nonwovens based on calcium alginate and chitosan can favor the formation of a network of weak bonds, such as hydrogen bonds and ionic bonds (salt bridges), with peptides. However, the direct use of BAC 9:1 nonwoven is limited by the esterification of chitin and, thus, the absence of free hydroxyl groups. Therefore, we used a nonwoven composed of 1:1:1 calcium alginate, chitosan, and chitin butyryl-acetyl co-polyester (BAC 9:1). The addition of hydrophobic BAC 9:1 is believed to improve the ability of calcium alginate and chitosan to interact with cells because cell membranes are hydrophobic in nature and can interact with hydrophobic substituents in the side chains of peptides **1**–**3**. Unfortunately, due to their high polarity, both calcium alginate and chitosan did not allow significant adhesion of cells to their surface. In order to overcome this obstacle, components such as polyethylene glycol, gelatin additives, and cross-linking agents are often used [93]. Adhesion of cells to solid materials can also be improved by using RGD peptides as additives [55,56,57,59,60,61,62,63].

Preparation of solid polysaccharide matrices modified with factors that can improve cell adhesion is time-consuming and also carries the risk of incomplete removal of reagents, which can affect the safety of the resulting materials. This can be overcome by using physical deposition methods such as dip-coating. However, the use of these methods is associated with the risk of irregular modifier deposition or accelerated release of the modifier in vitro and in vivo. In order to verify the uniformity of the deposition of RGD peptides on the polysaccharide matrices, an SEM analysis was performed used in this study. The results showed that the dip-coating method did not cause any changes in the material morphology (Figure 2). The images showed no visibly tighter fiber packages that could affect the ability of cells to interact with fibers or change the physicochemical and mechanical properties of nonwovens. In addition, there were no “sticky” areas where the fibrous structure of the material was disturbed. The results of the microscopic analyses confirmed the preserved ability of polysaccharide matrices with physically embedded RGD peptides to bind C2C12 cells. In all cases, we observed cells strongly adhered to nonwoven fibers. During muscle growth and repair, myoblasts fuse with each other generating multinucleated cells known as myotubes. One of the most important criteria of successful skeletal muscle bioengineering is the progression of cells to the stage of myotube formation. The elongated morphology of the C2C12 cells attached to the investigated nonwoven fibers suggests that the materials might improve the myogenesis process, confirming their potential usefulness as scaffolds in muscle regeneration. This, however, should be confirmed in future studies.

We also assessed the proliferative capacity of C2C12 cells and their morphology in the presence of RGD peptide-modified polysaccharide fibers. SEM and light microscopy with hematoxylin–eosin staining is widely used for evaluating cell morphology and adhesion to scaffold materials (both fibrous and porous) [94]. In this study, the release factor (Q) of RGD peptides (modifiers of solid polysaccharide matrices) was found to range from 10% to 100%, depending on both the structure of the solid polysaccharide matrices **A**–**C** and that of the RGD peptides **1**–**3**. The release factor is a key parameter in regenerative medicine, influencing the bioavailability of API in drugs and compounds affecting cell/tissue response. The determination of this parameter and understanding of its relationship with the properties of biologically active compounds may allow for the rational design of scaffolds and materials that can be used for tissue regeneration [95,96].

Structural and signaling responses of cells are governed by numerous adhesive interactions which involve adhesive receptors that selectively bind to external ligands. Adhesive receptors activate transmembrane signaling pathways, influencing cellular shape, dynamics, and fate. Integrins are a highly diverse class of adhesive receptors of ECM which perform basic biological functions in all higher organisms. The RGD sequence of fibronectin is the minimal integrin-binding motif and is also present in many other proteins. Thus far, many RGD-based peptide and nonpeptide ligands displaying varying degrees of specificity have been developed [97]. When bound to a solid support (insoluble form), RGD peptides can promote cell adhesion, whereas when these peptides come into contact with cells in a dissolved form, they inhibit cell adhesion [98]. Due to these properties and their very high release factor (Q), peptide **1** in polysaccharide matrices **A**–**C** may promote the inhibition of cell adhesion in the initial phase of incubation. For peptide **2**, a high release factor Q was observed only for conjugate C2, which indicates that conjugates of peptide **2** with polar matrix **A** or B allow improving cellular adhesion to polysaccharide fibers. Irrespective of the polysaccharide matrix used, cyclic peptide **3** showed a moderate release factor (Q), and thus, conjugates **A3**, **B3**, and **C3** could have positive effects on the cell adhesion process.

Given the current state of research on polysaccharide–peptide conjugates, it is not easy to identify the most optimal materials. In order to obtain polysaccharide–peptide materials with the desired cell adhesion-promoting effect, it is necessary to carefully select both the composition of the polysaccharide matrix (varies based on the content of polysaccharides in the nonwoven) and the mixture of RGD peptides. It should also be remembered that the different types of cells will have varying requirements for cell adhesion.

The formation of skeletal muscle fibers is a complex process. Chemical and biological interactions guide the formation of syncytial structures created by the fusion of myoblasts. The process of myoblast fusion is initiated by adhesion molecules. A previous study showed that the use of peptides containing RGD upregulated cell proliferation and decreased apoptosis [78]. In an in vitro study, McClure et al. [97] demonstrated the role of integrin alpha3, integrin beta1, ADAM12, CD9, CD81, M-cadherin, and VCAM-1 in muscle regeneration. A cascade of interactions is initialized by reactions occurring between ECM components and collagens I, III, and IV, as well as laminins, fibronectin, and proteoglycans. Integrin α5β1 binds fibronectin and simplifies the migration of myoblasts. As myoblasts differentiate into myocytes, integrin α5β1 is replaced by α7β1. During fiber formation, the presentation of this integrin increases, promoting myoblast fusion [99,100,101]. In the present study, we modified ECM by adding an RGD motif- containing biomimetic peptide analogs of tissue collagen to culture media and thus influenced only the first stage of the process of integrin–ECM interaction.

Although we observed relatively high rates of release of RGD peptides from the **A**–**C** matrices, our biological studies indicated that these materials are safe to use in the manufacture of scaffolds for regenerative medicine. An undoubted advantage of using chitosan-containing polysaccharide matrices in the composition of these scaffolds is their inherent antibacterial activity [90].

Muscle repair is a complicated and precise mechanism that involves three primary processes: removal of damaged cells, the proliferation of muscle stem cells (mainly satellite cells), and remodeling of damaged tissue. The most important challenge in muscle repair is modulating these processes to improve recovery. New materials used for modulation should significantly improve each stage of muscle regeneration and exhibit high biocompatibility. Therefore, it is essential to ensure that these materials have high functional properties and are nontoxic, nonsensitized, nongenotoxic, and nonirritating. In this study, we assessed the in vitro effect of three peptides on cell cycle and cell death, as well as their cytotoxicity and genotoxicity, to evaluate the potential negative effects of the newly developed materials on muscle satellite cells. We also analyzed how satellite cells grew around the material. The observed results are promising for further applied research.

## 5. Conclusions

We investigated the potential utility of peptide–polysaccharide conjugates as scaffolds for the regeneration of muscle tissue. Our study analyzed calcium alginate (**A**), chitosan (**B**), and a mixed nonwoven composed of equal amounts of calcium alginate, chitosan, and chitin butyryl-acetyl co-polyester (BAC 9:1) (**C**) as polysaccharide matrices, and H-RGDS-OH (**1**), H-GRGDS-NH_2_ (**2**), and a cyclo(RGDfC) (**3**) peptides containing the RGD motif. Peptide–polysaccharide conjugates were obtained by dip-coating, and the final derivatives contained both peptide and polysaccharide connected through a network of weak bonds. We found that the dip-coating application of peptides did not disturb the fibrous structure of the studied nonwovens. MTT and Ki-67 assays performed using the C2C12 cell line showed that the conjugates composed of calcium alginate (**A**) and the mixed polysaccharide nonwoven (**C**) had the best functional properties (no cytotoxicity or adverse effects on cell viability and proliferation ability). Peptides **1** and **2** were found to be the most optimal components, and their suitability for application in regenerative medicine was also confirmed by cytotoxicity assessments (LDH and Alamarblue^®^ assays), proliferation analysis using C2C12 cells (Ki-67 assay), and cell cycle analysis.

The ability of C2C12 cells to interact with the tested peptide–polysaccharide conjugates **A1**, **B1**, and **C1** was confirmed by SEM imaging and light microscopy with hematoxylin–eosin staining. We also tested the efficiency of release of peptides **1**–**3** from polysaccharide matrices **A**–**C** and found that the release efficiency differed depending on both the peptide and the polysaccharide matrix structures.

Peptide–polysaccharide conjugates containing chitosan (**B**) as a sugar component and nonwoven **C** were found to exhibit antibacterial activity against *S. aureus* and *K. pneumoniae*, which suggests that they can be classified as highly antibacterial materials. The most active antibacterial conjugates were those containing H-RGDS-OH (**1**) as their peptide. Our results indicate that the tested materials can be used to obtain scaffolds for the regeneration of damaged tissue with intrinsic antibacterial activity. This is particularly important from the perspective of increasing bacterial resistance to antibiotics.

Overall, the findings of this study show that the tested peptide–polysaccharide conjugates have potential applications as scaffolds in regenerative medicine.

## Figures and Tables

**Table 1:** Characteristics of the polysaccharide fibers.

Fiber	Length [mm]	Linear Density [dTex]	Tenacity [dTex]	Elongation at Break [%]
Calcium alginate	37.1	2.0	22.47	10.17
Chitosan	37.1	2.2	11.93	6.85
BAC 9:1	38.0	2.5	22.81	4.27

**Table 2:** Characteristics of the polysaccharide nonwovens **A**–**C** used as starting materials for the preparation of conjugates **A1**–**A3**, **B1**–**B3**, and **C1**–**C3**.

Non-Woven	Mass Per Square Meter [g/m^2^]	Thickness [mm]	Tensile Strength in the Longitudinal Direction [N]	Tensile Strength in the Transverse Direction [N]	Elongation in the Longitudinal Direction [%]	Elongation in the Transverse Direction [%]
**A** based on calcium alginate	98.8	1.9	16.72	25.32	88.90	47.65
**B** based on chitosan	116	2.1	17.23	26.70	88.39	47.29
**C** containing 1:1:1 calcium alginate:chitosan:BAC 9:1	120	2.1	17.35	25.90	86.40	46.62

**Table 3:** Characteristics of peptides **1**–**3** containing the RGD motif.

Peptide	HPLC, Retention Time [min]	HPLC, Purity [%]	Theoretical Weight	*m*/*z* Found
H-RGDS-OH (**1**)	2.85	95	433.19	434.23
H-GRGDS-NH_2_ (**2**)	2.34	98	489.23	490.23
Cyclo(RGDfC) (**3**)	3.65	97	579.22	578.23

**Table 4:** Antibacterial activity of the conjugates of polysaccharides **A**–**C** with peptides **1**–**3** against *Staphylococcus aureus* ATCC 6538.

Tested Material	*S. aureus* ATCC 6538		
	Value of Growth F (F = log C_T_ − log C_0_)	Value of Growth G (G = log T_T_ − log T_0_)	Value of Antibacterial Activity A (A = F − G)
**A1**	0.47	–0.31	0.78
**A2**	0.28	–0.18	0.46
**A3**	0.07	–0.04	0.11
**B1**	2.33	–1.53	3.87
**B2**	1.81	–1.19	3.01
**B3**	1.72	–1.13	2.85
**C1**	1.36	–0.90	2.26
**C2**	1.21	–0.80	2.01
**C3**	1.18	–0.78	1.96

C_T_—number of bacteria in the control sample after incubation; C_0_—number of bacteria in the control sample before incubation; T_T_—number of bacteria in tested materials after incubation; T_0_—number of bacteria in tested materials before incubation.

**Table 5:** Antibacterial activity of the conjugates of polysaccharides **A**–**C** with peptides **1**–**3** against *Klebsiella pneumoniae ATCC 4352*.

Tested Material	*K. pneumoniae ATCC 4352*		
	Value of Growth F (F = log C_T_ − log C_0_)	Value of Growth G (G = log T_T_ − log T_0_)	Value of Antibacterial Activity A (A = F − G)
**A1**	0.21	−0.13	0.34
**A2**	0.14	−0.09	0.23
**A3**	0.06	−0.04	0.10
**B1**	3.57	−2.35	5.92
**B2**	2.75	−1.81	4.56
**B3**	2.40	−1.58	3.98
**C1**	1.85	−1.22	3.07
**C2**	1.72	−1.14	2.86
**C3**	1.32	−0.87	2.19

C_T_—number of bacteria in the control sample after incubation; C_0_—number of bacteria in the control sample before incubation; T_T_—number of bacteria in tested materials after incubation; T_0_—number of bacteria in tested materials before incubation.

**Table 6:** Characteristics of peptides **1**–**3**, parameters of active substances.

Peptide Properties	Peptide		
	H-RGDS-OH (1)	H-GRGDS-NH_2_ (2)	cyclo(RGDfC) (3)
Polar surface area	270.05	304.94	283.5
AlogP	−7.1896	−8.7574	−2.4137
Hydrogen acceptor count	10	10	9
Hydrogen donor count	10	11	10

## Data Availability

Not applicable.

## Supplementary Materials

The following supporting information can be downloaded at: https://www.mdpi.com/article/10.3390/ma15186432/s1, Figure S1: HPLC spectra of H-RGDS-OH (1); Figure S2: MS spectra of H-RGDS-OH (**1**); Figure S3: HPLC spectra of H-GRGDS-NH2 (2); Figure S4: MS spectra of H-GRGDS-NH2 (2); Figure S5: HPLC spectra of linear precursor of **3**; Figure S6: MS spectra of the linear precursor of 3; Figure S7: HPLC spectra of cyclo(RGDfC) (3); Figure S8: MS spectra of cyclo(RGDfC) (3); Figure S9: Network of possible weak interactions between polysaccharide matrices **A**–**C** and peptides **1**–**3**; Figure S10: Release profiles for unmodified and modified dosage forms; Figure S11: Diagram of the test stand and research method; Figure S12: Standard curves of peptides 1–3; Figure S13: Summary of cell viability in the presence of peptides **1**–**3** (LDH assay). Results are presented as the percentage of the control values (mean ± SD). Each assay was performed in triplicate (*n* = 12).; Table S1: Summary of polysaccharide–peptide conjugates used in the study; Table S2: Symbols, markings, and morphological parameters of the tested samples; Table S3: Types of correlation equations for peptides **1**–**3**; Table S4: Correlation equations for conjugates **A1**–**A3**, **B1**–**B3**, and **C1**–**C3**.
