# Supplementary material for: Evaluation of Polysaccharide–Peptide Conjugates Containing the RGD Motif for Potential Use in Muscle Tissue Regeneration
Source: Materials (Basel). 2022 Sep 16;15(18):6432. doi: 10.3390/ma15186432 (PMC9503514; doi:10.3390/ma15186432)
Supplement: Supplementary file 1 [file materials-15-06432-s001.zip › materials-1881828-supplementary.pdf]

materials

Evaluation of Polysaccharide–Peptide Conjugates Containing the RGD Motif for Potential Use in Muscle Tissue Regeneration

Marcin Kolasa[1], Katarzyna Czerczak[2], Justyna Fraczyk[2], Lukasz Szymanski[3], Slawomir Lewicki[3], Anna Bednarowicz[4], Nina Tarzynska[4], Dominik Sikorski[4], Grzegorz Szparaga[4], Zbigniew Draczynski[4], Szczepan Cierniak[5], Urszula Brzoskowska[5], Grzegorz Galita[6], Ireneusz Majsterek[6], Dorota Bociaga[7], Paulina Krol[8] and Beata Kolesinska[2,*]

[1]Military Institute of Hygiene and Epidemiology, Department of Pharmacology and Toxicology, Kozielska 4, 01-163 Warsaw, Poland

[2]Institute of Organic Chemistry, Faculty of Chemistry, Lodz University of Technology, Zeromskiego 116, 90-924 Lodz, Poland

[3]Department of Molecular Biology, Institute of Genetics and Animal Biotechnology, Polish Academy of Science, Postępu 36A, 05-552, Magdalenka, Poland

[4]Institute of Material Sciences of Textiles and Polymer Composites, Faculty of Material Technologies and Textile Design, Lodz University of Technology, Zeromskiego 116, 90-924 Lodz, Poland

[5]Military Institute of Medicine, Szaserow 128, 04-141 Warsaw, Poland

[6]Department of Clinical Chemistry and Biochemistry, Medical University of Lodz, Narutowicza 60, 90-136 Lodz, Poland

[7]Institute of Materials Science and Engineering, Lodz University of Technology, Stefanowskiego 1/15, 90-537 Lodz, Poland

[8]Lukasiewicz Research Network-Textile Research Institute, Brzezinska 5/15, 92-103 Lodz, Poland

*Correspondence: beata.kolesinska@p.lodz.pl; Tel.: 48 42 631 32 61 .

General procedures

Functionalization (incorporation of the C-terminal amino acid) on chloro(2′-chloro)trityl resin

The chloro(2-chloro)trityl resin (1 g, 1.0 mmol/g) was pre-treated with CH_2Cl_2 (DCM) for 15–30 min, after which the solvent was filtered off. The resin was treated with a protected amino acid (1 equiv. in relation to resins) and diisopropylethylamine (DIPEA) (4 equiv. in relation to the resins) in CH_2Cl_2 (10 mL per 1 g resin). The suspension was shaken gently at room temperature for 2–4 h. In the next step, the solution was removed, and the resin was washed three times with CH_2Cl_2 (5 mL), twice with DMF (5 mL), once with a mixture of DCM : MeOH : DIPEA (17:2:1) (10 mL) and then three times with CH_2Cl_2 (5 mL). The functionalized resins were dried to constant weight in a desiccator.

Functionalization of Rink-amide resin

Rink amide resin (1 g, 1.0 mmol/g) was swelled in DCM for 15–30 min. The Fmoc protecting group was removed using 25% piperidine solution in DMF (2 × 5 min). In the next step, the resin was filtered off and washed three times with DCM (5 mL), twice with DMF (5 mL), and three times with DCM (5 mL). The previously obtained Fmoc-protected amino acid triazine ester, NMM (2 equiv.) and a catalytic amount of DMAP were added to the reactor. The suspension was shaken gently for 24 h at room temperature. In the next step, the resin was filtered off and washed three times with DCM (5 mL), twice with DMF (5 mL), and three times with DCM (5 mL). The functionalized resins were dried to constant weight in a desiccator.

Fmoc-deprotection

The Fmoc protecting group was removed using 25% piperidine solution in DMF (2 × 5 min).

Activation of the carboxyl function of amino acids while extending the peptide chain

Activation of the carboxyl function was carried out at room temperature. To a solution containing 3 equiv. of DMT/NMM/$TosO^-$ in DMF (10 mL) was added 3 equiv. of the corresponding Fmoc-protected amino acid, and NMM (6 equiv.). The reagent mixture was used directly for the next stage of the synthesis on resin.

Loading functionalized resins

The loading was determined based on absorbance measurements in the range of 295–310 nm. The absorbance value was read at 301 nm. Blank and test samples were prepared in parallel, as follows:

1. Preparation blank – to a 10 mL volumetric flask was added 1.6 mL MeOH + 0.4 mL piperidine made up to the desired volume with DCM.
2. Sample preparation – a resin aliquot (about 3–4 mg) was placed in a 10 mL volumetric flask to which 0.4 mL DCM and 0.4 mL piperidine were added. After 30 min, 1.6 mL each of MeOH and DCM were added to the set volume.
3. Resin loading was calculated based on the formula

$$mmol/g = A_{301}/7800 \times 10/m_{resin}\ [g]$$

Cleavage of peptides from resin

The peptidyl-resin was treated using a TFA/Et_3SiH/H_2O mixture with a volume ratio of 95 : 2.5 : 2.5 (about 20 mL/1 g resin) for 4 h with vigorous stirring of the suspension. The resin was then filtered off and the filtrate was evaporated using a stream of nitrogen gas. To the residue was added Et_2O (5 mL), cooled to 0–5°C. After peptide precipitation, centrifugation, and decantation of the ether phase, the residue was dissolved in water and lyophilized.

Peptide synthesis

Synthesis of H-RGDS-OH (1)

The synthesis was performed according to the procedures described above. For the first stage: chlorotrityl resin (1.0 g, 1.0 mmol), Fmoc-Ser(tBu)-OH (1.151 g, 3.0 mmol), DIPEA (6 mmol, 1.05 mL) were used. For the next reaction steps Fmoc-Asp(OtBu)-OH (1.234 g, 3.0 mmol), Fmoc-Gly-OH (0.892 g, 3.0 mmol), Fmoc-Arg(Pbf)-OH (1.946 g, 3.0 mmol), DMT/NMM/$TosO^-$ (1.239 g, 3.0 mmol) and NMM (0.66 mL, 6.0 mmol) were used.

HPLC: *t*R 2.85 min, purity = 95%. LC-MS: 434.2295 ($[M + H]^+$, $C_{15}H_{28}N_7O_8^+$; calc. 433.19).

Synthesis of H-GRGDS-NH_2 (2)

The synthesis was performed according to the procedures described above. For the first stage: Rink amide resin (1.0 g, 0.7 mmol), Fmoc-Ser(tBu)-OH (0.805 g, 2.1 mmol), DMT/NMM/$TosO^-$ (0.826 g, 2.1 mmol), NMM (2.1 mmol, 0.23 mL) were used. For the next reaction steps Fmoc-Asp(OtBu)-OH (0.864 g, 2.1 mmol), Fmoc-Gly-OH (0.624 g, 2.1 mmol), Fmoc-Arg(Pbf)-OH (1.362 g, 2.1 mmol), DMT/NMM/$TosO^-$ (0.903 g, 2.1 mmol) and NMM (0.46 mL, 4.2 mmol).

HPLC: *t*R 2.34 min, purity = 98%.

LC-MS): 490.2285 ($[M + H]^+$, $C_{17}H_{31}N_9O_8^+$; calc. 489.23).

Synthesis of cyclo(RGDfC) (3)

Linear precursor: H_2N-Asp(OtBu)D-Phe-Cys(Trt)-Arg(Pbf)Gly-OH. Starting materials for each coupling: chlorotrityl resin (1.0 g, 1.0 mmol), Fmoc-Gly-OH (0.892 g, 3.0 mmol), Fmoc-Arg(Pbf)-OH (1.946 g, 3.0 mmol), Fmoc-Cys(Trt)-OH (1.757 g, 3.0 mmol),

Fmoc-D-Phe-OH (1.162 g, 3.0 mmol), Fmoc-Asp(OtBu)-OH (1.234 g, 3.0 mmol), DMT/NMM/TosO$^-$ (1.239 g, 3.0 mmol), NMM (0.66 mL, 6.0 mmol). The peptide was cleaved from the resin using 50% triisopropylsilane (TIS) in dichloromethane. High Performance Liquid Chromatography (HPLC) (15–95% A in 30 min): *t*R 25.7 min, purity = 98%. Liquid Chromatography Mass Spectrometry (LC/MS): 1147.4739 ([M + H]$^+$, $C_{60}H_{74}N_8O_{12}S_2^+$; calc. 1146.45).

Cyclization: To a solution of DIPEA (162 µL, 0.9 mmol) in dichloromethane (200 mL) was added dropwise HATU (114 mg, 0.3 mmol) with HOBt (40.5 mg, 0.3 mmol) and the linear precursor (350 mg, 0.3 mmol) and DIPEA (54 µL). Dropwise addition was performed over 2 h. After vigorous stirring, the solution was left for 24 h. After confirming complete conversion of the peptide, the mixture was concentrated to 50 mL. The solution was washed with water (30 mL), 1 M $NaHSO_4$ (30 mL), water (30 mL), 1 M $NaHCO_3$ (30 mL), and again with water (30 mL). After removal of the organic solvent, deprotection was performed analogously to the standard procedure used for cleavage from the resin.

HPLC: *t*R 3.65 min, purity = 97%.

LC-MS: 579.2256 ([M + H]$^+$, $C_{24}H_{34}N_8O_7S^+$; calc. 578.23).

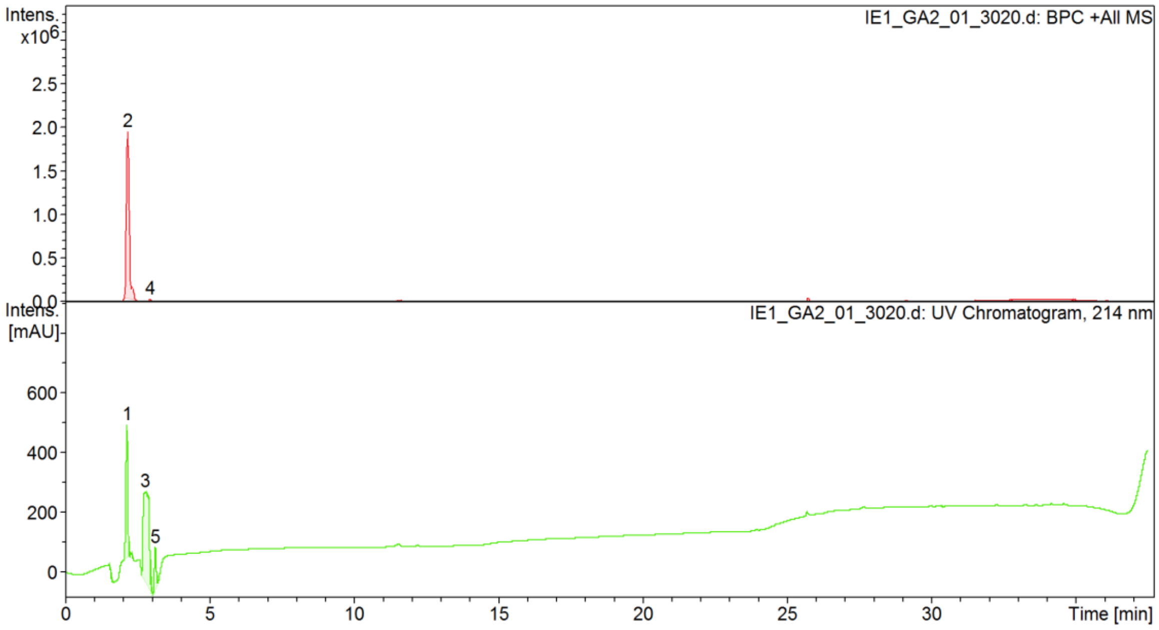

Figure S1. HPLC spectra of H-RGDS-OH adapted from [1].

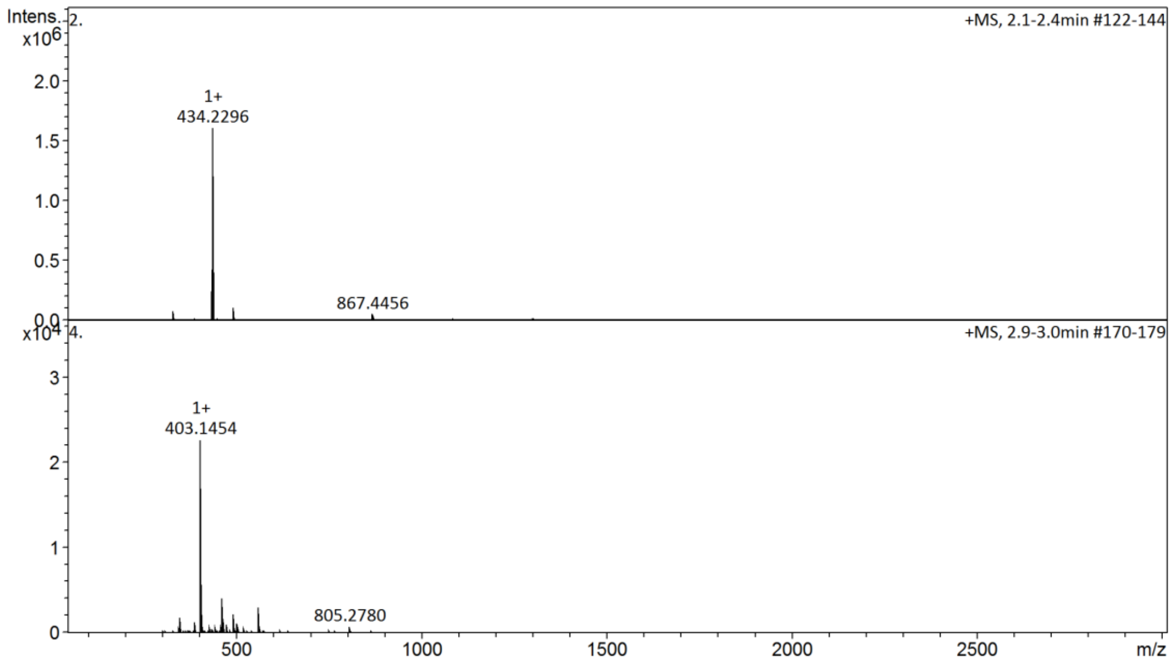

Figure S2. MS spectra of H-RGDS-OH (**1**).

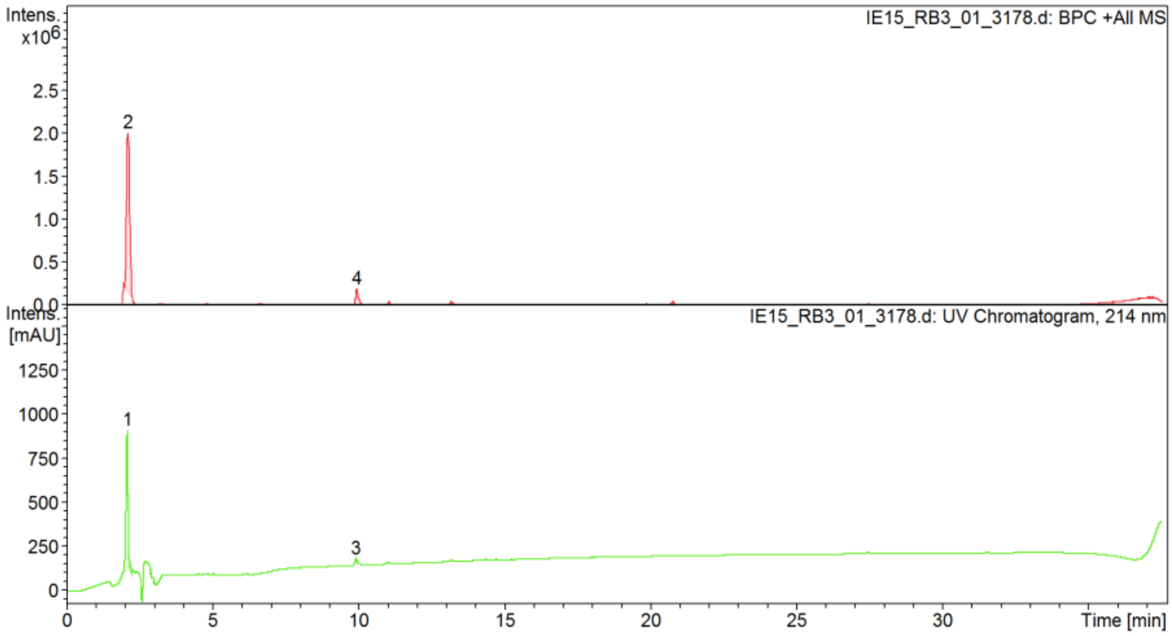

Figure S3. HPLC spectra of H-GRGDS-NH_2 (**2**).

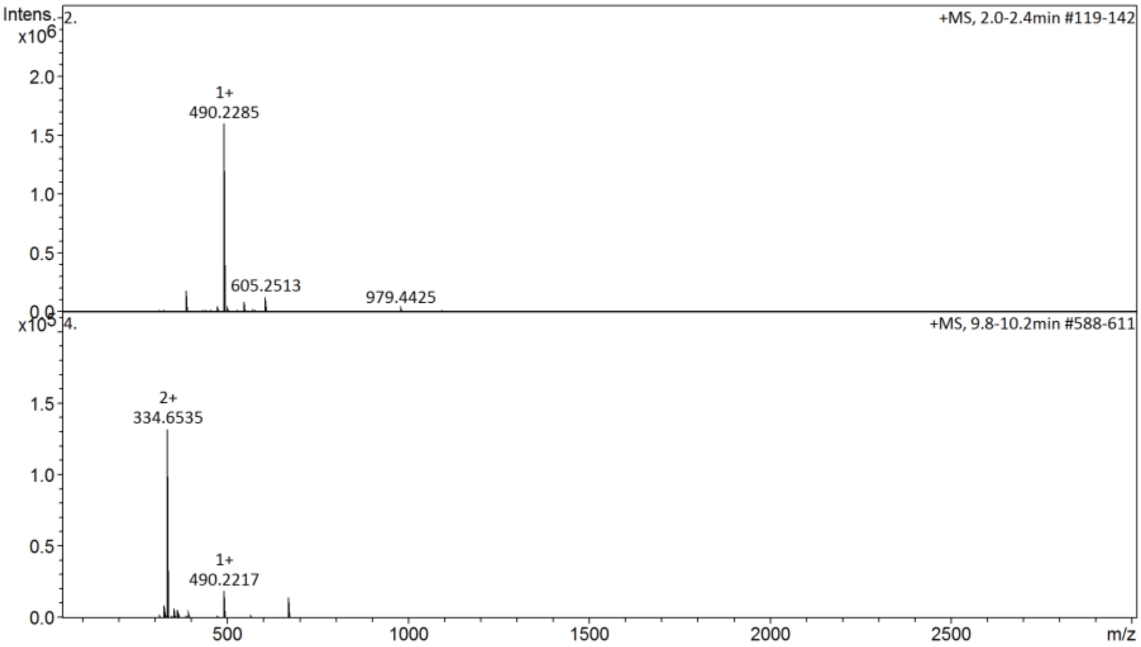

Figure S4. MS spectra of H-GRGDS-NH_2 (**2**).

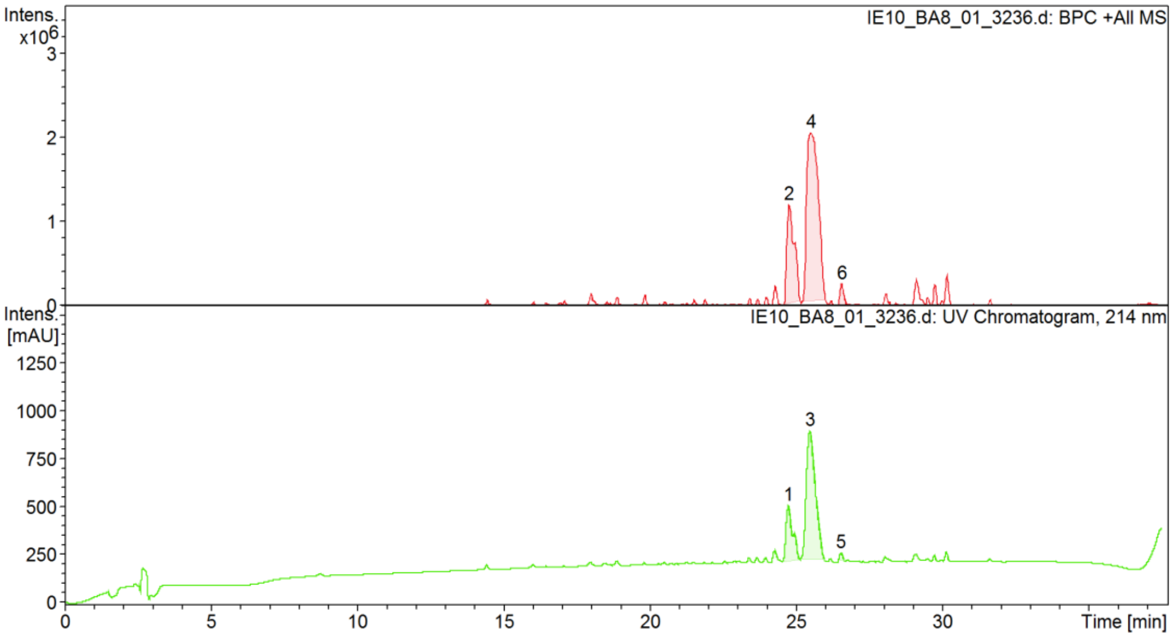

Figure S5. HPLC spectra of linear precursor of **3**.

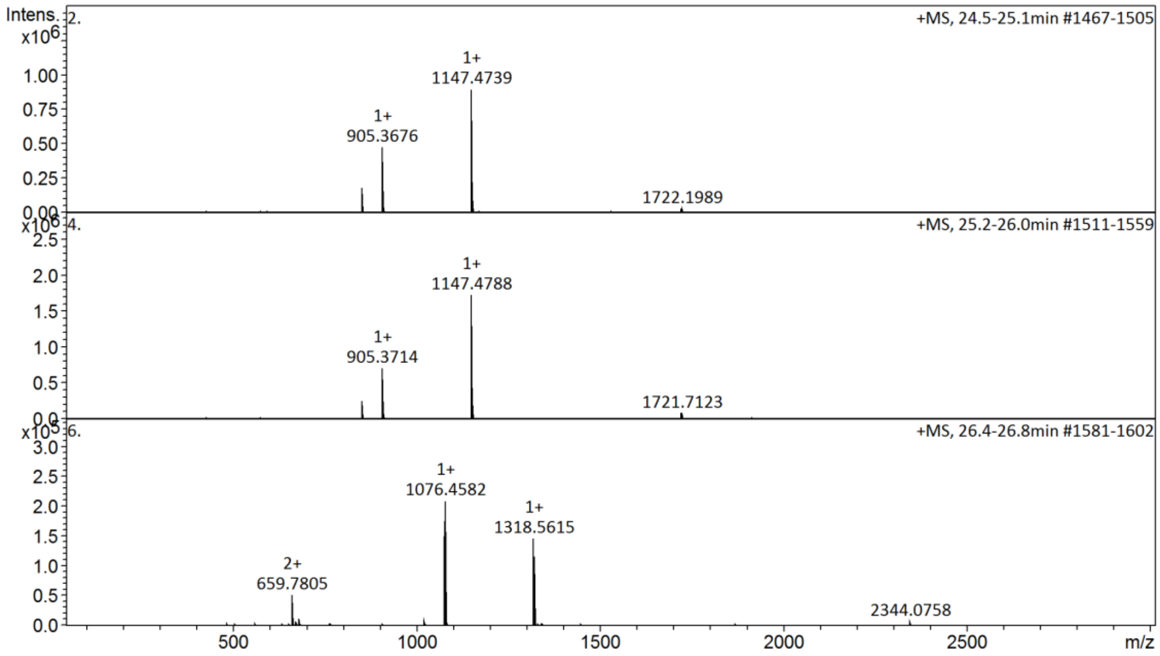

Figure S6. MS spectra of linear precursor of **3**.

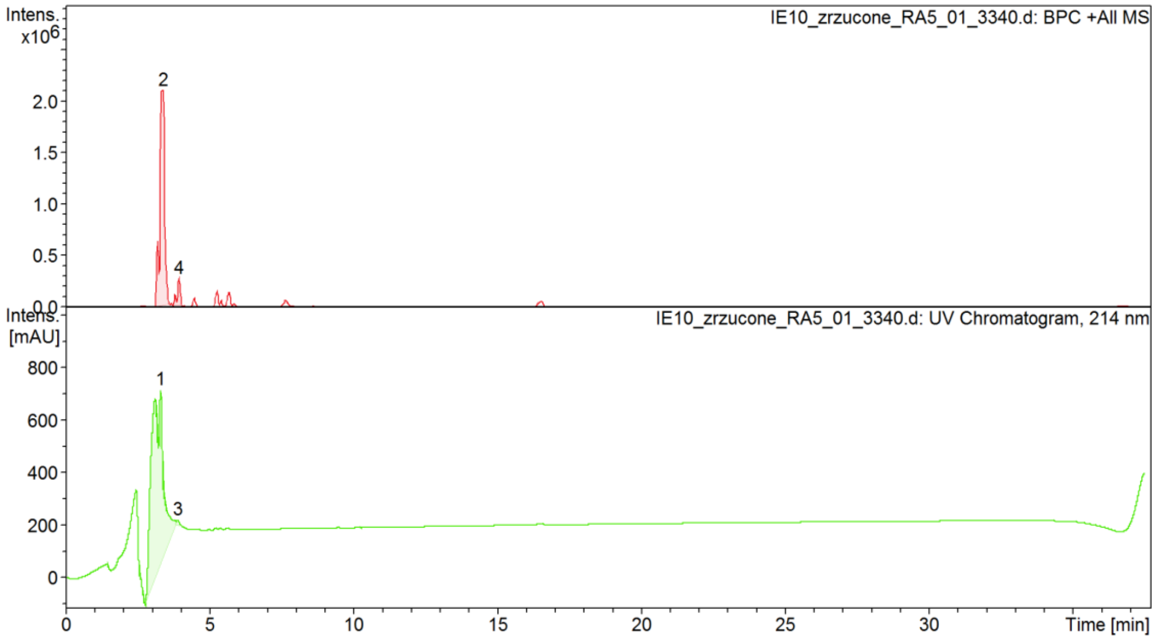

Figure S7. HPLC spectra of cyclo(RGDfC) (**3**).

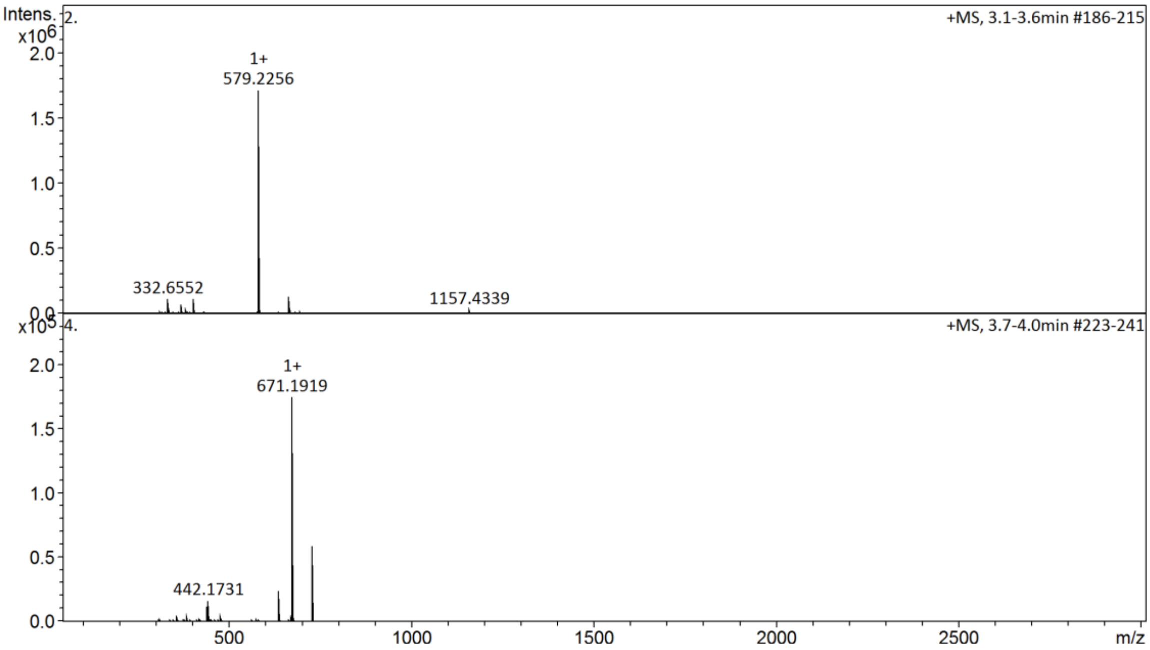

Figure S8. MS spectra of cyclo(RGDfC) (**3**).

Table S1. Summary of polysaccharide-peptide RGD conjugates used in the study.

Polysaccharide matrix	Peptide		
	**H-RGDS-OH (1)**	H-GRGDS-NH_2 (**2**)	cyclo(RGDfC) (**3**)
	Conjugate		
**calcium alginate (A)**	**A1**	**A2**	**A3**
chitosan (**B**)	**B1**	**B2**	**C3**
1:1:1 calcium alginate–chitosan– bur-tyryl-acetyl co-polyester of chitin (BAC 9:1) (**C**)	**C1**	**C2**	**C3**

a) alginate matrix (A)

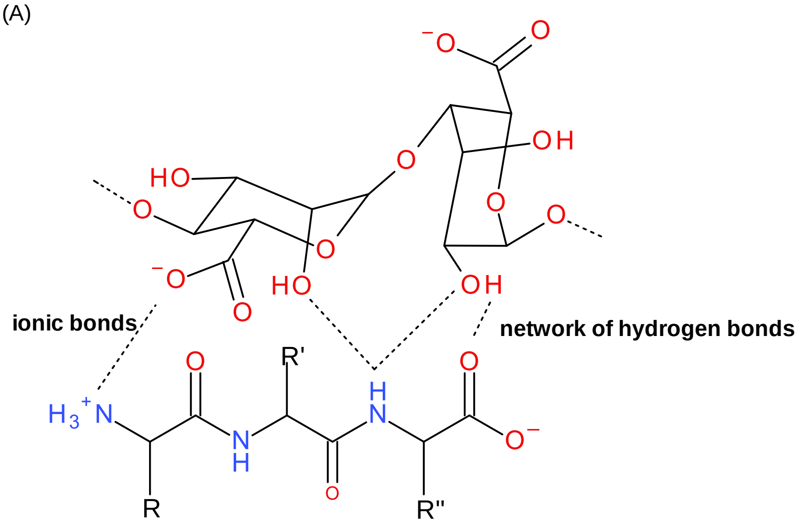

b) chitosan matrix (B)

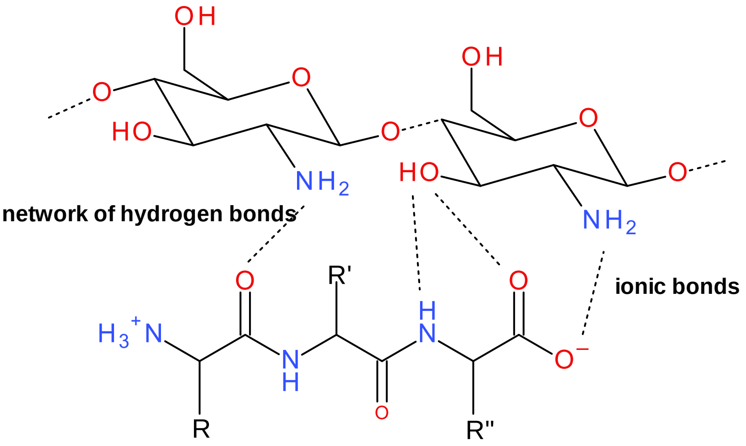

c) 1:1:1 calcium alginate - chitosan - burtyryl-acetyl co-polyester of chitin (BAC 9: 1) (matrix C)

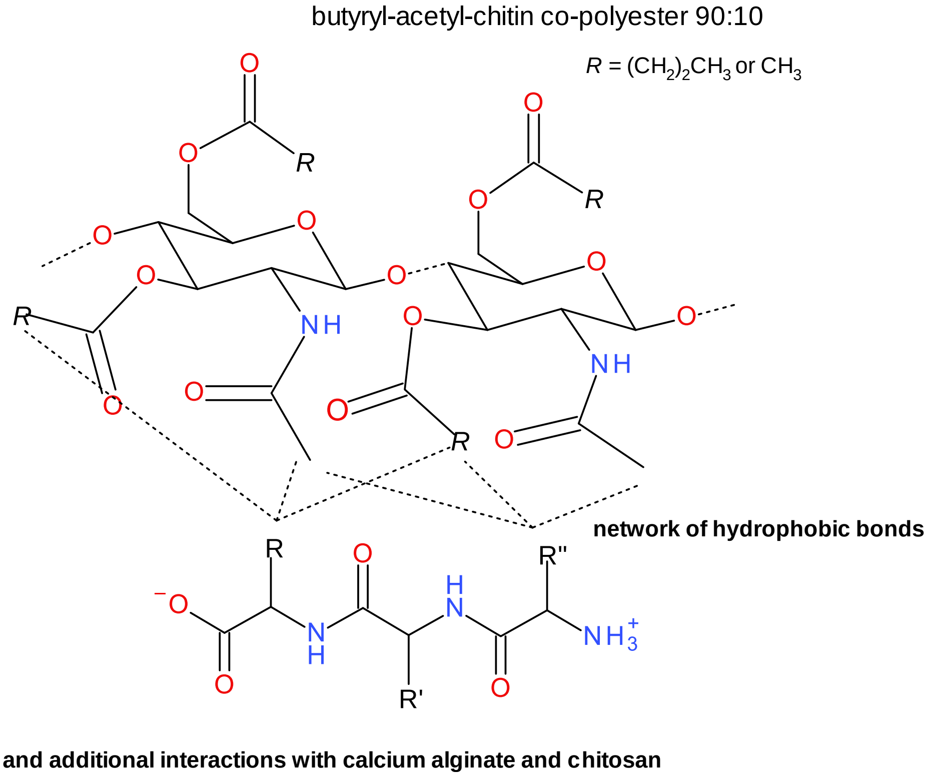

Figure S9. Network of possible weak interactions between polysaccharide matrices **A**–**C** and peptides **1**–**3**.

Release of peptides from non-woven substrates

Research methodology

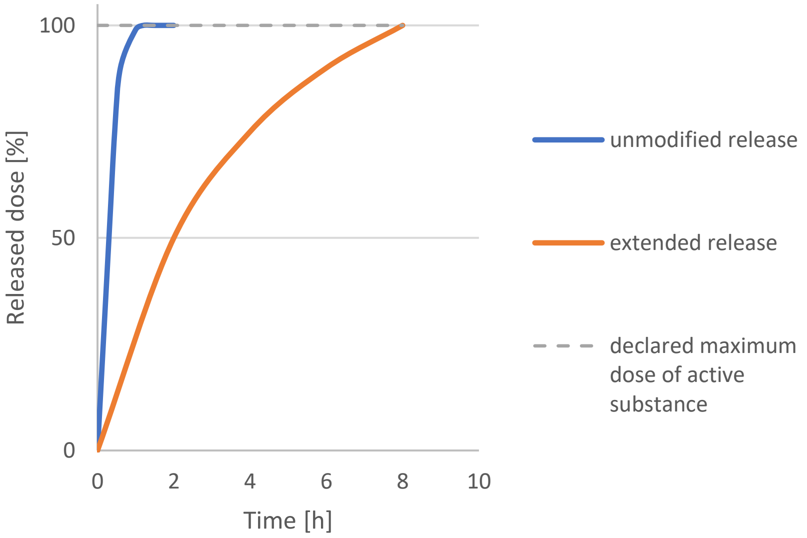

Figure S10. Release profiles for unmodified and modified dosage forms [1].

---

[1] *M. Sznitowska, Farmacja stosowana technologia postaci leku, PZWL, ISBN 97~3-200-5371-5, Warszawa, 2017*

Testing the release of active substances (peptides) from non-woven substrates, the so-called pharmaceutical availability study was carried out with a modified method involving the use of a magnetic stirrer with a heated plate instead of the basket method. This test was carried out for all obtained non-woven composites.

Table S2. Symbols, markings and morphological parameters of the tested samples.

Sample symbol	A1	A2	A3	B1	B2	B3	C1	C2	C3
Fiber type		alginate			chitosan			alginate-chitosan-butyryl acetyl chitin copolyester (BAC 9 : 1)	
Form of the material					needled non-woven fabric				
Peptide	1	2	3	1	2	3	1	2C	3
Surface weight of nonwovens		97 ± 5 g/m^2			141 ± 2 g/m^2			119 ± 19 g/m^2	
Thickness of nonwovens		2.61 ± 0.11 mm			2.07 ± 0.06 mm			1.88 ± 0.17 mm	
The mass of the tested sample, g	0.0216	0.0435	0.0325	0.0326	0.0273	0.0427	0.0354	0.0252	0.0187
The dimensions of the test sample					2 × 2 cm				
Maximum amount of peptide in the sample					2 mg/1cm^2 (approx. 8 mg in the tested sample)				

Phosphate buffer (PBS 1x concentrated) at 37°C was used as acceptor liquid. A magnetic stirrer was used to ensure continuous mixing of the sample, the sample was washed away with the acceptor fluid at a constant speed of 240 rpm. A fixed modulus was used that determined the amount of acceptor liquid in relation to the sample mass, which was 1: 1000 (sample mass: liquid volume). Samples with dimensions of approx. 2 × 2 cm, with the modulus of 1 : 1000, were flooded with acceptor liquid at 37°C, and kept at a constant temperature of 37°C using a water bath. Samples were taken at the appropriate time intervals: ½ minutes, ½ hours, 1 hour, 1½ hours, 2 hours, 2½ hours, 3 hours, 4 hours, 5 hours, 6 hours and 24 hours, keeping the amount of fluid constant and topping up the amount of fluid acceptor by the amount taken for testing (Figure S11).

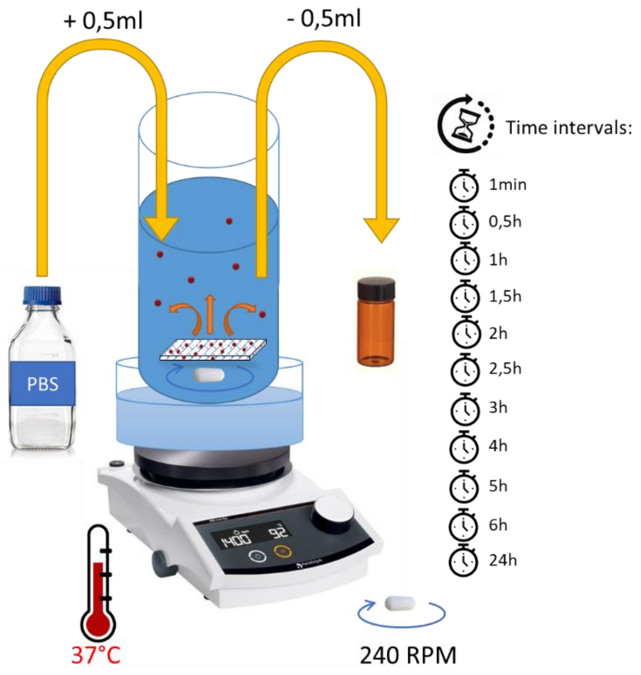

Figure S11. Diagram of the test stand and the method of conducting research.

Solutions containing 7.99 mg; 8.13 mg and 4.89 mg in 5mL PBS for peptides 1, 2 and 3, respectively. Dilutions (6 samples) were prepared from the solutions prepared in this way. The solutions were examined spectrophotometrically using the Thermo Scientific Orion AquaMate 8000 UV-Vis spectrophotometer using the VISION Lite Scan software in the wavelength range 190 - 660 nm. For the evaluation of the release, the absorbance value at the wavelengths of 202.5 nm, 202.6 nm and 202.4 nm for peptides **1**, **2** and **3**, respectively, was taken into account.

Table S3. Types of correlation equations for individual peptides **1**-**3**.

Peptide	Linerar regression	Exponential regression	Logarithmic regression	Power law regression	Wavelength λ
1	$y = 0.0393x + 0.0016$	$y = 0.0035e^{3.8582x}$	$y = 0.0076\ln(x) + 0.0245$	$y = 0.0369 \times ^{0.821}$	202.5 nm
	$R^2 = 0.9916$	$R^2 = 0.8977$	$R^2 = 0.9115$	$R^2 = 0.9968$	
2	$y = 0.0332x – 0.0031$	$y = 0.0022e^{2.7799x}$	$y = 0.0143\ln(x) + 0.0272$	$y = 0.0318 \times ^{1.3619}$	202.6 nm
	$R^2 = 0.9952$	$R^2 = 0.8707$	$R^2 = 0.8819$	$R^2 = 0.9925$	
3	$y = 0.0318x – 0.0032$	$y = 0.0012e^{4.9009x}$	$y = 0.0104\ln(x) + 0.0196$	$y = 0.0422 \times ^{1.6549}$	202.4 nm
	$R^2 = 0.9918$	$R^2 = 0.9054$	$R^2 = 0.988$	$R^2 = 0.9726$	

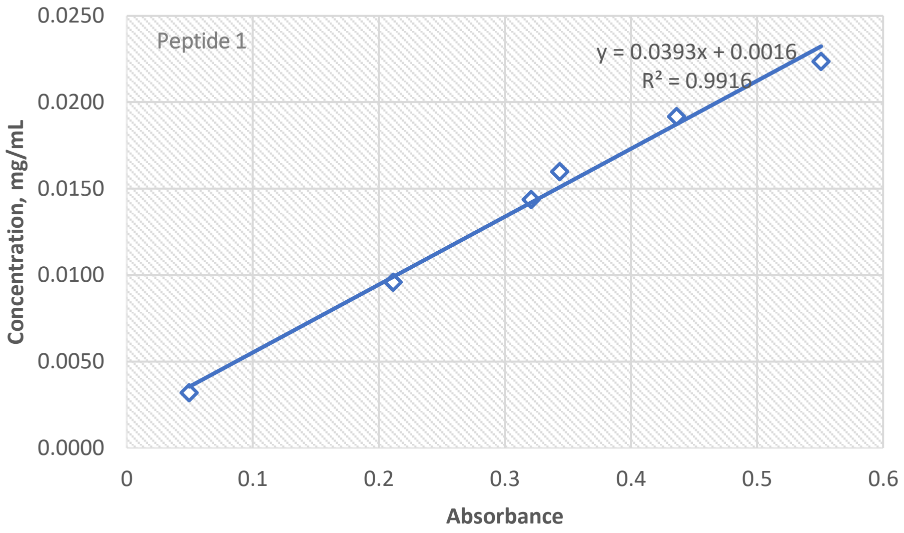

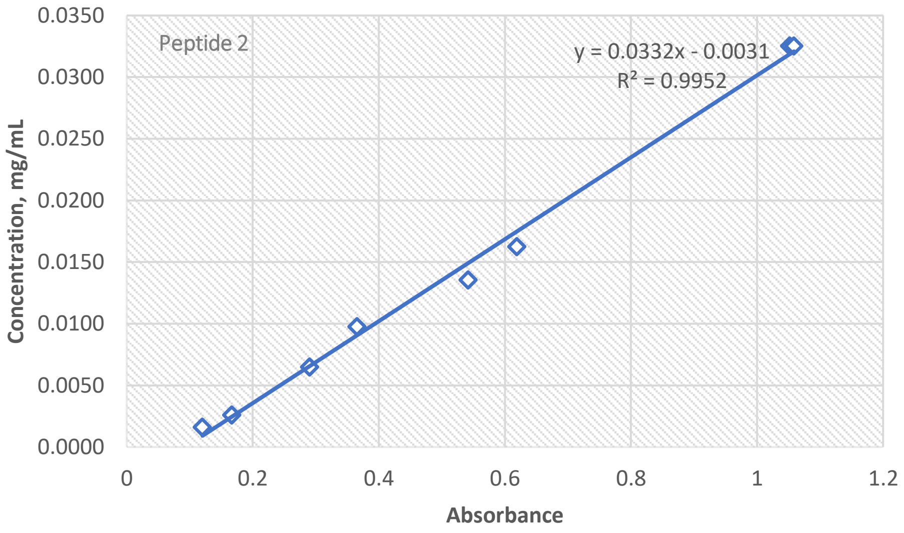

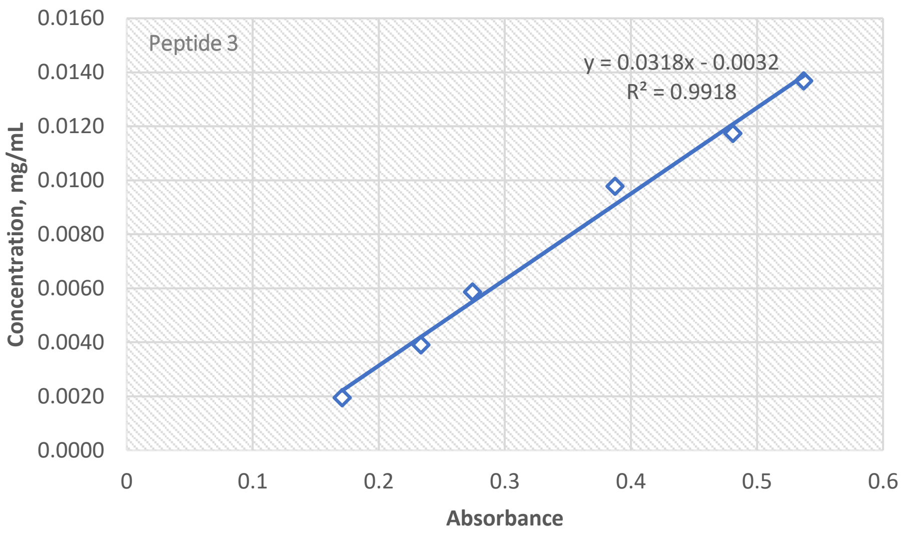

Figure S12. Standard curves of peptides **1-3**.

Table S4. Correlation equations for conjugates **A1–A3**, **B1–B3**, and **C1–C3**.

Conjugate	Linear regression	Exponential regression	Logarithmic regression	Power law regression	Wavelength λ
**A1**	$y = 2.2632x + 55.565$<br>$R^2 = 0.3808$	$y = 48.533e^{0.0406x}$<br>$R^2 = 0.224$	$y = 12.844\ln(x) + 59.423$<br>$R^2 = 0.9218$	$y = 50.06 \times {}^{0.3041}$<br>$R^2 = 0.9442$	202.5 nm
**A2**	$0.6788x + 5.3722$<br>$R^2 = 0.6545$	$y = 3.5684e^{0.096x}$<br>$R^2 = 0.2604$	$y = 2.5851\ln(x) + 7.1098$<br>$R^2 = 0.7315$	$y = 3.8235 \times {}^{0.666}$<br>$R^2 = 0.9653$	202.6 nm
**A3**	$y = 1.2942x + 23.681$<br>$R^2 = 0.3985$	$y = 18.873e^{0.0558x}$<br>$R^2 = 0.2162$	$y = 6.9615\ln(x) + 25.525$<br>$R^2 = 0.8698$	$y = 19.034 \times {}^{0.4241}$<br>$R^2 = 0.9427$	202.4 nm
**B1**	$y = 5.0664x + 63.647$<br>$R^2 = 0.5176$	$y = 53.288e^{0.0635x}$<br>$R^2 = 0.3323$	$y = 22.471\ln(x) + 73.601$<br>$R^2 = 0.7681$	$y = 56.941 \times {}^{0.3841}$<br>$R^2 = 0.9162$	202.5 nm
**B2**	$y = 0.1713x + 1.8914$<br>$R^2 = 0.5189$	$y = 1.1597e^{0.0908x}$<br>$R^2 = 0.1875$	$y = 0.7689\ln(x) + 2.2227$<br>$R^2 = 0.7888$	$y = 1.1442 \times {}^{0.7379}$<br>$R^2 = 0.9347$	202.6 nm
**B3**	$y = 0.9889x + 10.861$<br>$R^2 = 0.4556$	$y = 8.8127e^{0.0695x}$<br>$R^2 = 0.3572$	$y = 4.2105\ln(x) + 12.904$<br>$R^2 = 0.6231$	$y = 9.7517 \times {}^{0.3698}$<br>$R^2 = 0.7632$	202.4 nm
**C1**	$y = 5.2474x + 60.298$<br>$R^2 = 0.5141$	$y = 48.515e^{0.0694x}$<br>$R^2 = 0.3258$	$y = 23.051\ln(x) + 70.735$<br>$R^2 = 0.7482$	$y = 52.103 \times {}^{0.4215}$<br>$R^2 = 0.9063$	202.5 nm
**C2**	$y = 2.9622x + 32.474$<br>$R^2 = 0.5423$	$y = 26.38e^{0.0704x}$<br>$R^2 = 0.3368$	$y = 12.691\ln(x) + 38.55$<br>$R^2 = 0.7508$	$y = 28.438 \times {}^{0.4226}$<br>$R^2 = 0.916$	202.6 nm
**C3**	$y = 1.3168x + 10.76$<br>$R^2 = 0.5525$	$y = 6.3367e^{0.1046x}$<br>$R^2 = 0.2564$	$y = 5.4773\ln(x) + 13.555$<br>$R^2 = 0.7211$	$y = 6.6714 \times {}^{0.733}$<br>$R^2 = 0.9502$	202.4 nm

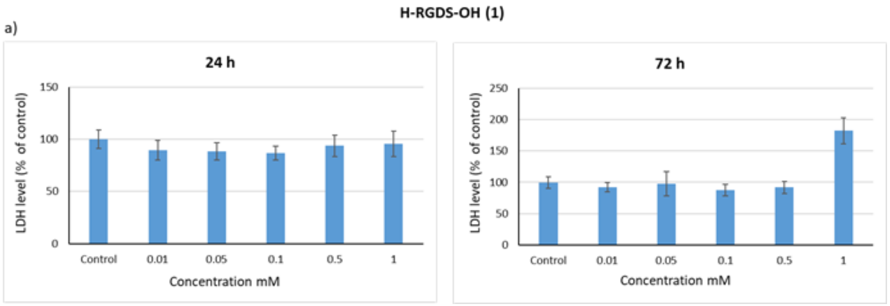

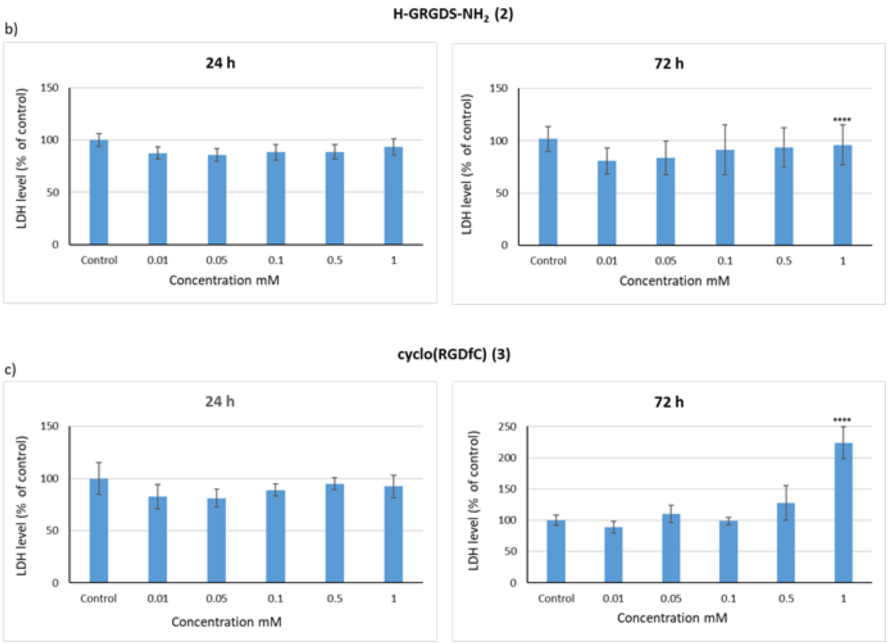

Figure S13. Summary of cell viability in the presence of peptides **1**–**3**, LDH assay. The results are presented as the percentage of the control values (mean ± SD). Each assay was performed in triplicate (n = 12).
